# *Caenorhabditis elegans* exhibits positive gravitaxis

**DOI:** 10.1186/s12915-021-01119-9

**Published:** 2021-09-14

**Authors:** Wei-Long Chen, Hungtang Ko, Han-Sheng Chuang, David M. Raizen, Haim H. Bau

**Affiliations:** 1 Department of Mechanical Engineering and Applied Mechanics, University of Pennsylvania, Philadelphia, PA USA; 2 Department of Biomedical Engineering, National Cheng Kung University (NCKU), Tainan, Taiwan; 3 Department of Neurology, Perelman School of Medicine, University of Pennsylvania, Philadelphia, PA USA; 4 Current Address: School of Mechanical Engineering, Georgia Institute of Technology, Atlanta, GA USA

**Keywords:** *Caenorhabditis elegans*, Gravity, Taxis behavior, Dopamine, Sensory function, Cilia, Gravitaxis

## Abstract

**Background:**

Gravity plays an important role in most life forms on Earth. Yet, a complete molecular understanding of sensing and responding to gravity is lacking. While there are anatomical differences among animals, there is a remarkable conservation across phylogeny at the molecular level. *Caenorhabditis elegans* is suitable for gene discovery approaches that may help identify molecular mechanisms of gravity sensing. It is unknown whether *C. elegans* can sense the direction of gravity.

**Results:**

In aqueous solutions, motile *C. elegans* nematodes align their swimming direction with the gravity vector direction while immobile worms do not. The worms orient downward regardless of whether they are suspended in a solution less dense (downward sedimentation) or denser (upward sedimentation) than themselves. Gravitaxis is minimally affected by the animals’ gait but requires sensory cilia and dopamine neurotransmission, as well as motility; it does not require genes that function in the body touch response.

**Conclusions:**

Gravitaxis is not mediated by passive forces such as non-uniform mass distribution or hydrodynamic effects. Rather, it is mediated by active neural processes that involve sensory cilia and dopamine. *C*. *elegans* provides a genetically tractable system to study molecular and neural mechanisms of gravity sensing.

**Supplementary Information:**

The online version contains supplementary material available at 10.1186/s12915-021-01119-9.

## Background

Gravity plays an important role in most life forms on Earth, ranging from single cells to plants [1] and animals. Aquatic invertebrates use gravity cues to help navigate [2]. Both terrestrial and aquatic vertebrates know which direction is up. While there are gravity sensory organ differences that relate to the unique ecologies across phylogeny, there are also similarities in the anatomical and physiological principles of such organs. Many molecular components of sensing and responding to gravity remain unknown. Understanding how animals respond to gravity would be aided by using organisms amenable to high-throughput genetic discovery approaches. Our focus here is on the nematode *Caenorhabditis elegans* (*C*. *elegans*), a model organism that has proven powerful for the molecular genetic dissection of other sensory modalities including olfaction, gustation, and mechanosensation,

Our interest in whether *C. elegans* senses and responds to gravity was triggered by observations that *C. elegans* swims in water-filled conduits. Since *C. elegans* is heavier than water, one would expect it to sink to the conduit’s bottom unless it were able to sense the direction of gravity and adjust its swimming direction to negate gravitational settling. Careful observations [3] have revealed that the animals do, indeed, sink to the bottom and interact with the bottom surface through frequent collisions (bumps). The question of whether *C. elegans* can sense and respond to gravity has remained unanswered.

Since *C. elegans* is heavier than the buffers typically used in laboratory experiments, it settles to the bottom of the vessel when suspended in such solutions [3]. Our observations suggest that as worms settle, they orient their direction of swimming to align with the direction of the gravity vector. Here, we examine the mechanisms responsible for the worm’s response to gravity. We show that this response is not passive, i.e., it is not mediated by factors such as non-uniform mass distribution and/or hydrodynamic effects. Rather, our experiments suggest that it is mediated by the animal’s nervous system. *C. elegans* offers important experimental advantages to unravel the molecular mechanisms of gravity sensing, including a small and simple nervous system, accessibility to rapid genetic manipulation, and ease of cultivation. What we learn in such studies is likely to be of significance to other animals, including humans.

## Results

**Fig. 1:**
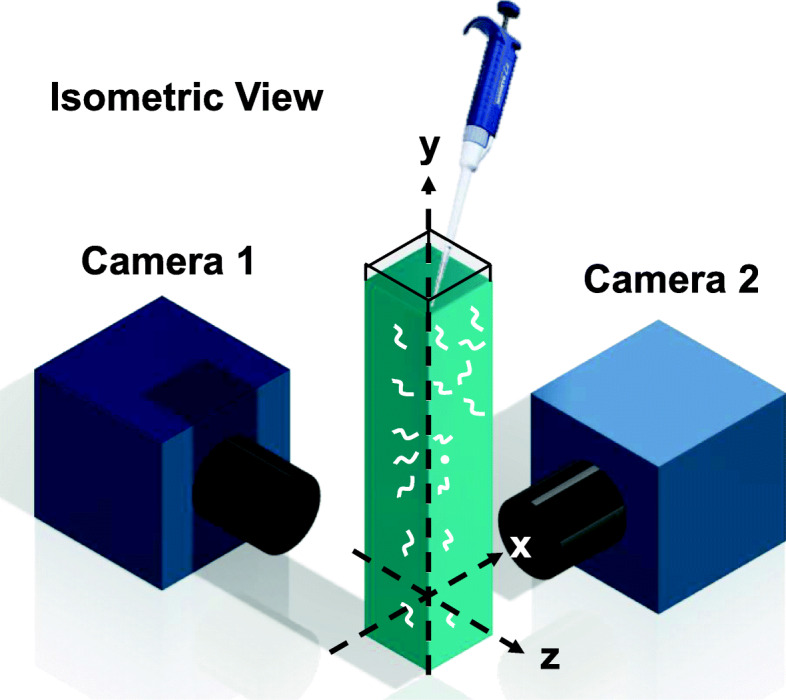
Experimental set-up (isometric view)

We released animals beneath the M9 buffer surface in a cuvette and monitored with two cameras the animals’ polar (*θ*) and azimuthal *(φ*) angles as functions of time and position beneath the liquid surface (Fig. 1). *θ* = 180° is the direction of gravity. Since the worms (~ 1.07 g/mL [4]) are heavier than the M9 buffer (~ 1 g/mL), they settled to the cuvette’s bottom.

### Well-fed wild-type *C. elegans* young adults align their swimming direction with the direction of gravity

**Fig. 2:**
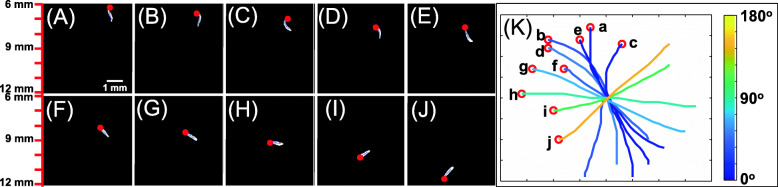
Wild-type animals rotate to align their direction of motion as they descend in solution. **A**–**J** 10 images collected at 1 image per second of a descending young adult, wild-type worm. The red dot indicates the position of the worm’s head. The animals are 6–12 mm beneath the water surface. The polar angle varies from 5.1° (frame **A**) to 141.5° (frame **J**). **K** The skeletons of the worm from **A**–**J** are depicted in colors corresponding to their angles of decent and shifted to align their geometric centers

As time went by, the wild-type worms varied their swimming direction to align with the direction of the gravity vector. Figure 2 depicts time-lapsed frames, 1 s apart, of a young adult animal settling in our cuvette. In the first image (A), the animal was ~ 6.5 mm beneath the water surface and faces nearly upwards *θ ~* 5°. As time elapsed, the polar angle *θ* gradually increased. In the last frame (J), the animal was ~ 11.5 mm beneath the water surface and its polar angle was *θ*~142°. Thus, in a 10-s period, the worm changed its swimming direction from nearly upwards to nearly downwards.

This behavior is exhibited more clearly in panel K, wherein we colored the skeletons to correspond with their angle of decent and translated the skeletons of the animal to align their centroids. The animal rotated to align its direction of swimming with the direction of gravity. We observed this same behavior numerous times in two different labs (one in Pennsylvania and one in Taiwan, Fig. S4), indicating that this is a reproducible behavior. The orienting behavior was independent of the animal’s azimuthal position (Fig. S5).

**Fig. 3:**
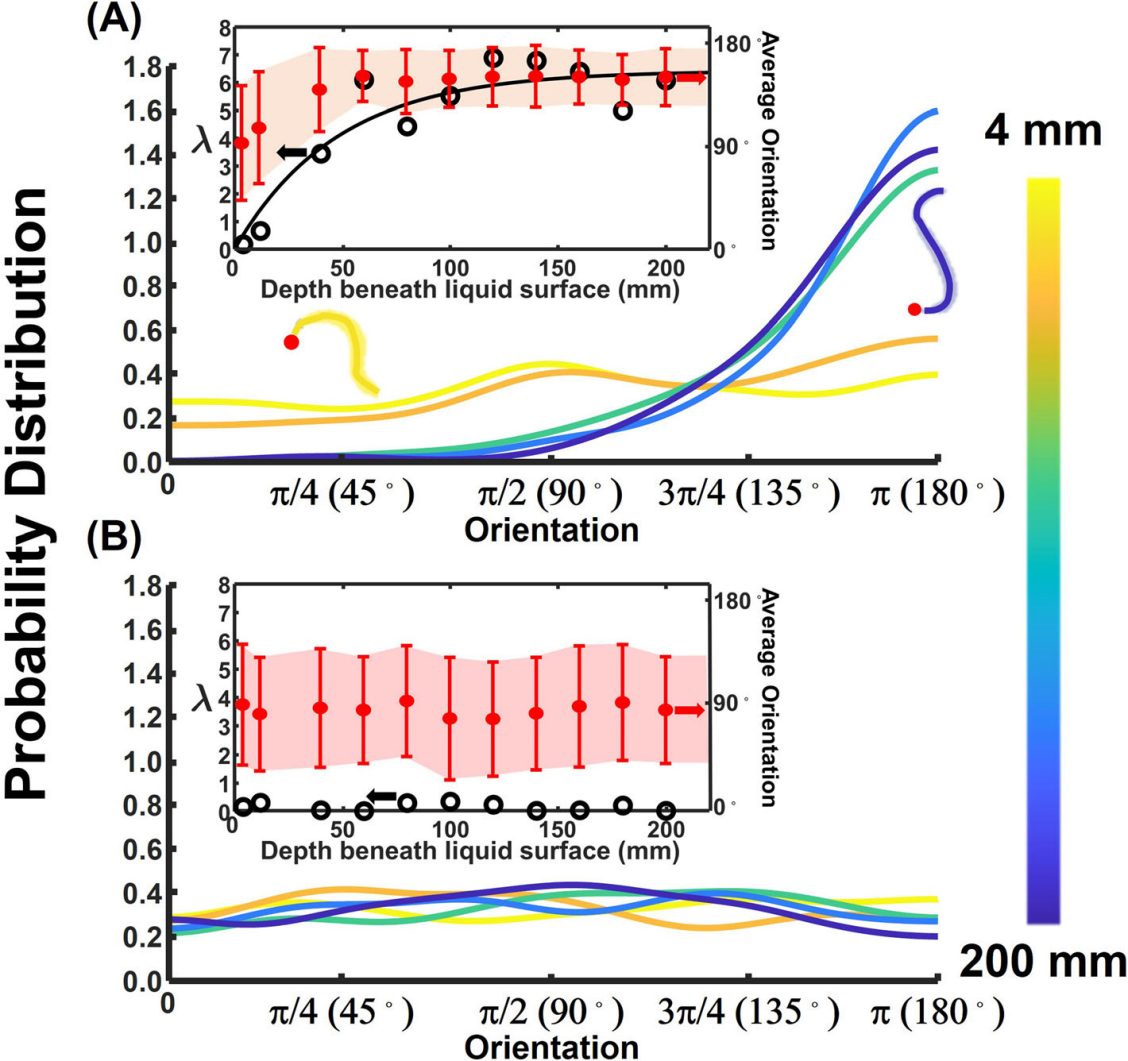
Wild-type worms change their orientation as they settle in solution whereas immobile worms do not. **A** Kernel (probability) density estimate (KDE) of wild-type swimmers’ orientation angle (*θ*) at positions 4 mm (*N* = 145), 12 mm (*N* = 141), 80 mm (*N* = 132), 140 mm (*N* = 122), and 200 mm (*N* = 124), beneath the liquid surface. **B** KDE of heat-shocked paralyzed WT animals at positions 4 mm (*N* = 121), 12 mm (*N* = 131), 80 mm (*N* = 129), 140 mm (*N* = 128), and 200 mm (*N* = 132) beneath the liquid surface. The figure was produced with the MATLAB^TM^ function “ksdensity” with “bandwidth” of *π*/12. The animals’ cartoons depict the average orientation of the animal with the animal’s color indicating the animal’s position beneath the liquid surface and the red dot indicating the animal’s head. Insets depict the concentration parameter *λ* on the left vertical axis (hollow circles) and the average orientation angle (± one standard deviation) on the right vertical axis (solid circles) as functions of the animal’s position beneath the surface. Additional data is provided in ESI Figs. S10 and S11

The kernel density estimate KDE *f(θ)* (an approximation of the probability distribution function, *pdf*) [5] of the animals’ orientations at various depths beneath the liquid surface (Fig. 3) resembles the von Mises-Fisher directional *pdf* on a sphere [6] centered about the downward direction (*θ* = 180^o^) (SI-Sections S6):

1 $$ f\left(\theta \right)=\frac{\lambda }{2 Sinh\lambda}{e}^{\lambda \cos \left(\pi -\theta \right)}\sin \left(\pi -\theta \right), $$

where the *concentration parameter λ* (reciprocal measure of dispersion) is analogous to the inverse of the variance in a normal distribution. *λ➔0* corresponds to a uniform distribution. When *λ* ≥ 1 and *λ* ≥ 3, over 73% and 95% of the animals are facing downwards (*θ* > 90°), respectively.

Close to the liquid’s surface (shortly after release), *f(θ)* is nearly symmetric about the horizontal direction (*θ* = 90°), indicating equal probability towards upward and downward swimming. As time increases, the KDE skews in the direction of increasing polar angles, the average orientation angle increases, and the scatter about the average polar angle decreases. As the worms descend, they rotate to increase their polar angle and align their direction of swimming with the direction of gravity.

We computed the concentration parameter *λ* for our data by fitting the cumulative distribution function (*cdf*) associated with Eq. 1 (Fig. S9) to the experimental *cdf*. When the wild-type animals were at depth *d* = 4 mm beneath the surface, *λ* ~ 0.18, reflecting a nearly uniform distribution. As the animals’ depth increased, the animals had more time to align with the direction of gravity, and the skewness of the KDE and the magnitude of *λ* increased.

2 $$ \lambda (d)\sim {\lambda}_{\infty}\left(1-{e}^{-\beta d}\right), $$

where *λ*_*∞*_ ~ 6.3 and *β* ~ 0.02 mm^−1^ (inset, Fig. 3A). KDEs at depths ranging between 120 and 200 mm nearly overlapped (ESI—Section S7), indicating that the animals have reached an equilibrium condition at a depth of ~ 120 mm. Similar behavior was observed in experiments carried out in glass tubes (Fig. S6), indicating that the observed behavior is not caused by the cuvette’s material.

**Fig. 4:**
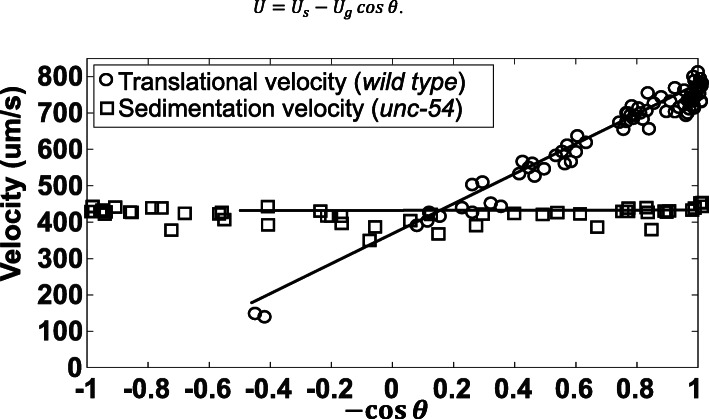
Swimming velocity and sedimentation velocity of worms during gravitaxis. Velocity of first day adult wild-type worms (*N* = 79) and of motion-impaired adult mutant *unc-54* worms (*N* = 52) as functions of −cos *θ*, where *θ* = 0 corresponds to upward orientation. Since *unc-54* does not swim, it only sediments. In contrast, the translational velocity of wild-type worms is a superposition of both sedimentation and swimming velocity (Eq. 3)

To correlate the animal’s depth with its residence time in solution, we examined the worm’s translational and angular velocities. The velocity (*U*) of young adult wild-type worms’ centroid in the direction of swimming depended nearly linearly (*R*^2^ = 0.89, solid line) on (− cos *θ*) (Fig. 4).

3 $$ U={U}_s-{U}_g\cos \theta . $$

We interpret *U*_*s*_ as the animal’s swimming velocity and *U*_*g*_ as its sedimentation velocity in the gravitational field. The term (−*U*_*g*_ cos θ) is the projection of the sedimentation velocity on the animal’s swimming direction. In contrast to rigid cylindrical rods [4] whose settling velocity varies with the polar angle, the worm’s settling velocity *U*_*g*_ depended only weakly on the orientation (*θ*)*.* This is perhaps because the worm is neither straight nor rigid. We estimate $\overline{U_s}\approx 411\ \upmu \mathrm{m}/\mathrm{s}$ and $\overline{U_g}\approx 432\ \upmu \mathrm{m}/\mathrm{s}$. The angular velocity $\omega =\frac{d\theta}{d t}$ varied widely, but never exceeded *28*°/s. In summary, wild-type animals align their swimming direction with the gravity vector. Is this a passive or active response to gravity?

### Paralyzed animals do not align with the gravity direction

To address this question, we monitored the orientation of sedimenting animals with severe movement impairment. We achieved muscle paralysis (or near paralysis) by three different methods: (A) exposing wild-type animals to an elevated temperature (heat shock), (B) immersing wild-type worms in sodium azide solution, and (C) testing animals that carry a mutation in the major muscle myosin gene *unc-54* [7]. We considered only fully paralyzed *unc-54* mutants (about 33% of the population). We expect paralyzed animals to have a mass distribution like that of wild-type motile control animals. This is most likely to be true in the case of the wild-type animals paralyzed with brief exposures to sodium azide because this paralysis is fully reversible after washing out the azide.

Paralyzed animals did not align with the direction of gravity during downward sedimentation (heat-shocked: SI-Fig. S11 and S12, 4 mm < *d* < 100 mm, and Fig. 3B, 4 mm < *d* < 200 mm; sodium azide-paralyzed animals: Fig. S13, *d* = 100 mm and *d* = 200 mm, and *unc-54*: Fig. S14, *d* = 40 mm). In all cases, the descent angle *θ* is nearly symmetric with respect to the horizontal plane (*θ* ~ 90^o^) and the orientation distribution does not vary with depth/residence time (*λ* of paralyzed animals ranged from 0.004 to 0.30, inset in Fig. 3B). Contrast Fig. 3B and Fig. S11 with Fig. 3A and Fig. S9. The differences are striking. Active WT animals rotate to align with the gravity vector while immobile animals do not.

The measured settling velocity (*U*_*g*_ ~ 432 μm/s) of the paralyzed worms (heat-shocked and *unc-54*) favorably agrees with the estimated contribution of gravitational settling to the velocity of the WT animals (Eq. 3 and Fig. 4). Since the paralyzed animals have similar diameter and length as the wild-type animals, similar settling velocities indicate that the paralyzed animals’ masses are like those of wild-type worms. Furthermore, the lack of rotation of paralyzed animals suggests that the animals are not significantly top heavy and that there are no convective currents in our experimental apparatus.

### Upward-sedimenting wild-type animals gravitax

Might the hydrodynamic interaction between the flow field induced by the animal’s swimming gait and the flow field associated with the animal’s settling rotate the animal to align it with the direction of gravity? We reasoned that if the downward swimming orientation was the result of interactions between the flow field induced by the swimmer and the flow field associated with downward settling, then symmetry considerations would require that animals sedimenting *upwards* would align with the up direction and exhibit negative gravitaxis. To test this hypothesis, we suspended well-fed wild-type animals in a homogeneous LUDOX suspension that was slightly denser than the animals. Our experiments were complicated by the animals floating to the surface, providing a relatively short time for observations, and by the LUDOX suspension having viscosity greater than water, decreasing the rotational velocity of the animals. These two factors resulted in less time to orient in the gravitational field.

**Fig. 5:**
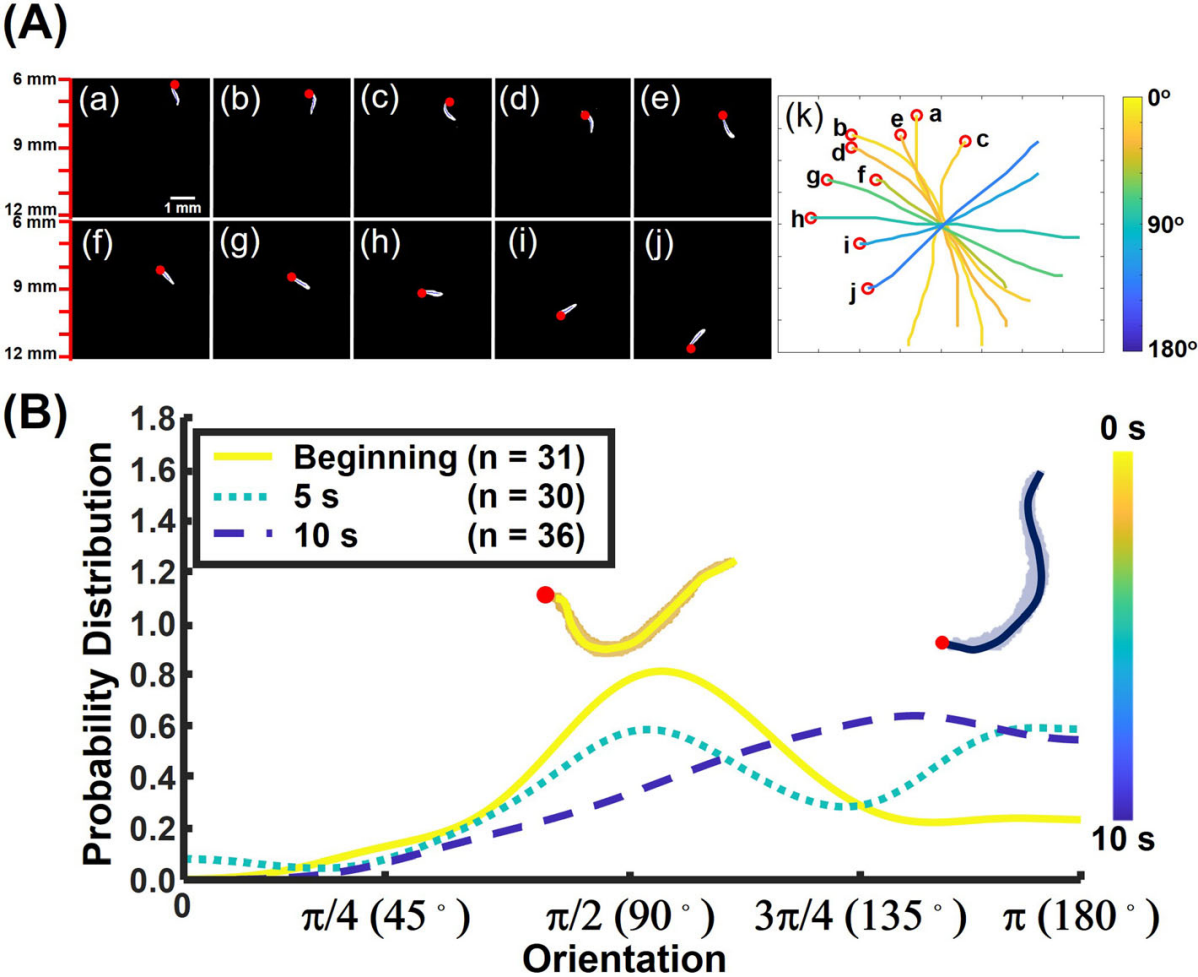
Wild-type animals rotate to align their direction of motion downward when suspended in a solution denser than the animals. **A** (a–j) 10 images collected at 1 image per second of a young adult, wild-type worm. The red dot indicates the position of the worm’s head. The polar angle varied from 49° in frame (a) to 138.9° in frame (j). (k) The skeletons of the worms in a–j were shifted to align their geometric centers. **B** The kernel (probability) density estimates of the orientation angle of animals suspended in LUDOX HS-40 suspension (density 1.1 g/mL and viscosity about 7 times that of water) beginning < 2 s after the animals were introduced into the suspension, 5 s later, and 10s later. *N*_0_ = 31, *N*_5s_ = 30, and *N*_10s_ = 36. In depicting the KDE curves, we used Matlab^TM^ default values. The cartoons depict the average orientation of the animals at the color-coded time

Notwithstanding these experimental complications, upward floating animals still rotated to orient downwards and swim in the direction of the gravity vector. Figure 5A (a–j) shows 10 images, captured at a rate of 1 per second, of a young adult, well-fed, wild-type worm. The red dot indicates the position of the worm’s head. In the 10-s period of observation, the polar angle increased from the initial value of 49° in frame a to 139° in frame j. Frame k depicts the skeletons of the worms from frames a–j shifted to align their geometric centers, illustrating the animal’s tendency to align with the direction of gravity.

At short times, the KDE (Fig. 5B) resembled a *sin* function, characteristic of a uniform *pdf* in *θ* (random orientation). As time passed, the peak of the KDE shifted to larger values of the polar angle *θ* and the magnitude of the concentration factor *λ* increased from near 0 to 2.7, consistent with the animals adjusting their orientation to align with the direction of gravity.

**Fig. 6:**
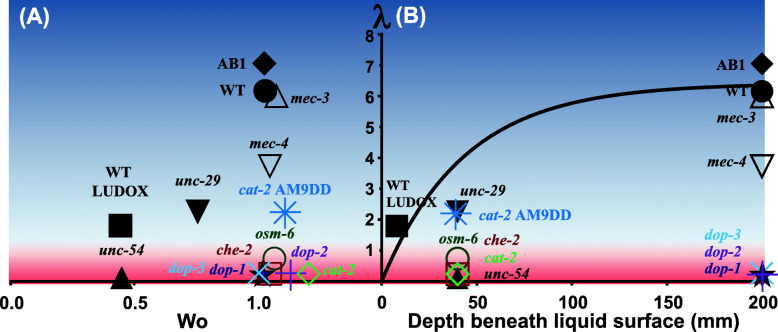
Animals’ propensity to gravitax (*λ*) as a function of animal vitality (*Wo*) (**A**) and the animal’s depth beneath the liquid surface (**B**). The reddish region (*λ* < 1) indicates the absence of gravitaxis and an orientational probability distribution (pdf) that is dissimilar to that of wild-type animals with a confidence level greater than 95%. The bluish region indicates competency to gravitax (*λ* > 1). The solid line in **B** corresponds to Eq. (2) for N2. The residence time of animals in LUDOX was converted to equivalent depth with the aid of the settling velocity

In summary, animals suspended in a liquid that is either lighter (Fig. 3) or heavier (Fig. 6) than themselves rotate to swim in the direction of the gravity vector. Our observation of downward swimming even during upward sedimentation suggests that gravitaxis is not explained by hydrodynamic interactions.

### Starved animals and animals mildly defective in muscle function (*unc-29*) align with the direction of the gravity vector, albeit at a slower rate than well-fed WT animals

Is the propensity for positive gravitaxis behavior affected by the dietary history or by mild impairment (non-paralysis) in body movements? The angle of descent of starved (> 1 h from the last feeding) wild-type animals (Fig. S15) and *unc-29* mutants (Fig. S16), which are mildly defective in neuromuscular transmission due to a mutation in an acetylcholine receptor subunit [8], increased as the animals’ depth beneath the liquid surface (and residence time) increased. The concentration factor *λ* of the starved WT animals increased at about half the rate of the well-fed animals and that of *unc-29* mutants even more slowly (Fig. S17).

Importantly, even animals with reduced swimming vigor, given enough time, orient to align with the gravity vector. Positive gravitaxis behavior does not solely rely on vigorous muscle movements.

### Ciliated sensory neurons are required for gravitaxis

We reasoned that if the worm uses its nervous system to sense gravity and orient in the downward direction, we should be able to impair this behavior by disrupting sensation while minimally impairing movement. Many sensory functions of *C. elegans* are mediated by neurons that extend cilia to the nose of the animal. Is gravity sensation mediated by ciliated sensory neurons? To test this hypothesis, we analyzed the angle of descent of animals mutant for the gene *osm-6* or for the gene *che-2*, which encode, respectively, the intraflagellar proteins 52 (IFT52) and 80 (IFT80) and in which sensory cilia are globally disrupted [9].

The polar angles and concentration factor *λ* of the mutants *che-2* (*λ* ~ 0.16, *d* = 40 mm) and *osm-6* (*λ* ~ 0.66, *d* = 40 mm) did not vary as they descended. Their angles of descent were uniformly distributed with nearly equal probability for upward and downward swimming (Figs. S18 and S19). Our data suggest that ciliated sensory neuron function is required for gravitaxis.

### Deficient mechanosensation does not impair gravitaxis

In vertebrates, the mechanism of sensing gravity involves the transduction of mechanical stimuli caused by deflection of sensory cilia into a neuronal electrical signal [10]. We therefore considered the possibility that the *C. elegans* mechanosensory system is involved in sensing gravity. *C. elegans* hermaphrodites have 30 neurons, including both ciliated and non-ciliated neurons, with demonstrated mechanosensory function [11]. These include neurons that mediate response to soft or harsh touch to the body as well as neurons involved in response to touch to the head. Mutants in the gene *mec-4*, which encodes a DEG/ENaC cation channel, are defective in the function of six mechanosensory neurons responsible for the response to light touch to the body [11]. Mutants in the gene *mec-3*, which encodes a LIM-type homeodomain protein required for the proper development of the six mechanosensory neurons as well as for the PVD and FLP neurons, are additionally defective in response to harsh touch to the body [11].

Both *mec-4* and *mec-3* mutant animals responded to gravity, albeit perhaps not as strongly as the wild-type worms (Fig. S19). *mec-3* mutants were more defective in gravitaxis than *mec-4* mutants, but neither mutant was as defective as the *che-2* or *osm-6* mutants. Our results suggest that the body touch mechanosensory neurons are not strictly required for gravitaxis.

### Dopamine-deficient worms are defective in gravitaxis

Certain mechanosensory functions of *C. elegans* require dopaminergic neurotransmission [12]. To test the hypothesis that dopaminergic transmission plays a role in gravitaxis, we experimented with *cat-2* mutants. *cat-2* encodes a tyrosine hydroxylase, which catalyzes the conversion of tyrosine to L-DOPA, the chemical precursor for dopamine. *cat-2* mutant animals therefore have reduced levels of dopamine.

*cat-2(e1112)* mutants that carry an early stop mutation in the *cat-2* gene were severely defective in orientating with the direction of gravity (Fig. S20). To increase the likelihood that this phenotype is due to the mutation at the *cat-2* locus (rather than some other undefined background mutation), we tested an independent *cat-2* allele, *n4547*, which contains a 1007-bp deletion and is presumably a null mutant [13]. *n4547* mutants also showed defective orientation with the gravity vector (Fig. S20), supporting the conclusion that the *cat-2* gene is required for gravitaxis.

To test in another fashion whether dopaminergic neurotransmission is required for gravitaxis, we examined the settling behavior of the mutants for *dop-1*, *dop-2*, and *dop-3*, which encode dopamine receptors. All three dopamine receptor mutants did not align with the direction of gravity and had equal probability of swimming upwards as downwards (Fig. S20). These data suggest that the ability to synthesize and respond to dopamine is essential for gravitaxis.

### The gravitaxis defect of *cat-2* mutants is rescued pharmacologically

If the *cat-2* inability to synthesize dopamine endogenously does, indeed, cause deficiency in gravitaxis, then exogenous dopamine might rescue this defect. We carried out three sets of experiments to test this hypothesis. In the first set of experiments, dubbed “agar doped with dopamine (ADD),” we cultivated *cat-2* mutants for 65 h prior to our experiment on an agar plate doped with dopamine and then performed the experiment using regular M9 buffer. In the second set of experiments, dubbed “M9 buffer doped with dopamine (M9DD),” we cultivated *cat-2* mutants prior to the experiment on regular NGM agar plates and then performed the experiment in M9 buffer doped with dopamine. In the third set of experiments, dubbed “Agar and M9 Doped with Dopamine (AM9DD),” we cultivated *cat-2* mutants on a dopamine-doped agar and carried out the experiment in an M9 solution doped with dopamine. M9DD animals exhibited nearly uniform kernel density distribution, failed to gravitax, and descended with an average inclination angle of 88°±56° (*N* = 84) (Fig. S21). In contrast, ADD animals partially recovered their ability to respond to gravity and descended with an average descent angle of 113°±53° (*N* = 70). The contrast between the skewed kernel density function of the ADD animals (Fig. S21) and the uniform KDE of the untreated *cat-2* animals (Fig. S20) is striking. The doubly treated animals AM9DD further improved their ability to respond to gravity and descended with an average angle of descent of 133°±47° (*N* = 77). Therefore, pharmaceutical treatment with dopamine rescued *cat-2* mutant ability to react to gravitational forces. While the ADD experiment could be consistent with either functional or developmental roles for dopamine in gravitaxis, the AM9DD experiment, which showed the best rescue of the *cat-2* defect, supports the former.

### Mobile, gravitaxis-impaired animals do not differ significantly in their swimming gaits from gravitaxis-competent animals

Is it possible that differences in the various mutant animals’ swimming gaits are responsible for gravitaxis? We compared the swimming gaits of WT, *osm-6*, *cat-2*, and dopamine-treated *cat-2* (ESI-Section S14). While the swimming gaits of these worms are not identical, differences are small. These animals all swam at similar body bend frequencies and had similar body bending amplitudes (Figs S22-S24). Consistent with prior reports [13, 14], the gravitaxis-competent wild-type animals and the gravitaxis-incompetent *osm-6* mutants had similar swimming gaits. Importantly, differences in swim gaits between wild-type and gravitaxis-deficient animals were smaller than the difference between WT and *unc-29* mutants, which did show gravitaxis.

### Magnetotaxis is not responsible for the worm orientation in the gravitational field

Vidal-Gadea et al. [15] reported that *C. elegans* orients to the Earth’s magnetic field during vertical burrowing migrations. Well-fed adult worms of the N2 Bristol strain, which was isolated in the Northern Hemisphere, migrate up, while starved N2 worms migrate down [15]. In contrast, well-fed adult worms of the AB1 Adelaide strain, which was isolated in the Southern Hemisphere, migrate down, while starved AB1 worms migrate up in response to the same magnetic field.

We did not observe similar tendencies in our experiments. In our experiments, well-fed N2 (Figs. 2 and 3A), well-fed AB1 worms (Figs. S25 and S26), and starved (Figs. S15 and S17) N2 animals all oriented with the direction of the gravity vector and swam downwards. Hence, the taxis mechanism identified in [15] is unlikely to explain our observations.

### *C. elegans* on agar does not gravitax

Mechanosensory forces encountered by the worm are different between liquid and solid substrates. We repeated our experiments with WT animals and *mec-3* mutants placed on a vertical agar disk seeded with bacteria (SI Section S16). We included the *mec-3* mutants to minimize the contribution of body touch sensitivity to animal’s propensity to respond to gravity. We divided the disk into four quadrants—two above and two below the horizontal diameter and counted the number of wild-type animals (Fig. S28) and *mec-3* mutants (Fig. S29) in each quadrant at the beginning of the experiment and 12 h later.

Furthermore, we monitored the orientation of both animal types as functions of time. Both the wild-type control animals and *mec-3* mutants exhibited uniform distributions of orientations that did not significantly vary with time; the worms were nearly uniformly distributed among the quadrants both at the start and the end of the experiment, exhibiting no preference to migrate in the direction of gravity. To further minimize sensory input provided by the bacterial particles, we repeated the experiment in the absence of food. Food-deprived WT and *mec-3* (data not shown) exhibited similar orientational distributions as their well-fed counterparts. Our experiments suggest that worms on agar do not exhibit gravitaxis.

## Discussion

We monitored the responses of wild-type *C. elegans* and various mutant animals to Earth’s gravity under various conditions (Fig. 6 and Table S1 summarize our findings). *C. elegans* is heavier than water and settles in aqueous solution. When in liquid, both well-fed and starved Bristol N2, Adelaide AB1, *mec-3*, *mec-4*, *unc-29*, and *cat-2* pharmacologically treated with dopamine aligned with the direction of gravity. Animals lacking muscle function either due to induced paralysis (by heat shock or by sodium azide exposure) or genetic impairment (*unc-54*), animals with globally disrupted cilia (*osm-6* and *che-2*), and animals defective in dopamine biosynthesis or signaling (*cat-2*, *dop-1*, *dop-2*, and *dop-3*) did not align with the direction of gravity when suspended in liquid. Furthermore, Bristol N2 wild-type worms and *mec-3* mutants did not align with the direction of gravity when placed in a vertical agar plate.

We determined that the probability distribution functions of gravitaxis-capable, suspended animals and gravitaxis-deficient animals are statistically distinct with the Kolmogorov-Smirnov (KS) two-sample test (Table S3). The hypotheses that (A) the pdf of gravitaxis-incompetent animals is the same as that of wild-type N2 worms and (B) the pdf of gravitaxis-capable animals is the same as that of the heat-shocked animals at a given depth were both rejected with confidence exceeding 99% (Table S3).

In prior experimental and theoretical studies, we found that various behavioral propensities of *C. elegans* such as gait synchronization [16], swimming against the flow (rheotaxis) [17, 18], and swimming along surfaces (bordertaxis) [18] can be explained by simple mechanics. Thus, we first examine the obvious potential cause of gravitaxis: are the animals top heavy and if so, does top heaviness cause the animals to align with the direction of gravity?

### *C. elegans* is either not top heavy or, if it is, then its top heaviness does not cause alignment with the direction of gravity

The mass distribution along the *C. elegans* body length is not known. The animals store fat primarily in the intestine [19], which is not present in the anterior 1/5th of the worm’s body. In contrast, the pharynx, which is a large muscular organ, is found only in the anterior 1/5th of the worm. Hence, it is possible (but not certain) that the animals are top heavy. If non-uniform mass distribution were the sole cause of animal’s tendency to orient with the direction of gravity (gravitaxis), both mobile and immobile animals would align with the direction of gravity. Our experiments indicate otherwise. Animals paralyzed by heat shock (Fig. S11-S12), by exposure to sodium azide (Fig. S13), or by a mutation in muscle myosin (Fig. S14), all fail to align with the direction of gravity. Although it is theoretically possible that the mass distribution of heat-shocked wild-type animals and of *unc-54* mutants differs from that of wild-type *C. elegans*, such a deviation is far less likely in the azide-paralyzed animals since the azide paralysis is rapidly and fully reversible. Lack of gravitaxis in the dopamine-deficient mutant *cat-2* (Fig. S20) and the pharmacological rescue of *cat-2* (Fig. S21) further reduces the possibility that gravitaxis can be attributed to non-uniform mass distribution. It is unlikely that dopaminergic transmission would affect mass distribution. Taken together, our data suggests that *C. elegans* is either not top heavy, or, if it is top heavy, then its top heaviness does not cause alignment with the direction of gravity.

Indeed, top heaviness does not necessarily imply alignment with the direction of gravity. Hydrodynamics characterizes the behavior of moving objects based on the ratio between inertial and viscous forces—the Reynolds number *Re = U L/ν*, where *U* is the object’s speed, *L* is the object’s characteristic length, and *ν* is the kinematic viscosity of the suspending fluid. When inertia is negligible (*Re➔0*), slender objects with fore–aft symmetry sediment at the angle at which they had been released [4]. Objects with non-uniform mass distribution orient to bring their center of mass beneath their centroid. This is, however, not necessarily true when *Re* > 0.

When *Re* > 0, cylindrical objects with uniform mass distribution rotate to attain a horizontal (broadside, *θ* = 90°) posture and settle horizontally [20]. This tendency may persist even when the cylindrical object is somewhat top heavy so long as the resulting rotational torque is not large. We illustrate this behavior with a simple experiment (ESI Section S17), wherein we released a partially folded (top heavy) 100-μm diameter, 1-mm-long metal wire in our cuvette. Although top heavy, the wire sedimented nearly broadside, featuring a nearly flat kernel distribution estimate (Fig. S30) like our paralyzed animals (Fig. 3B). This metal wire experiment does not directly inform on nematode behavior. Although the wire settles at a higher Reynolds number than the nematode, it is subject to a much greater torque to align it with the direction of gravity. We describe this wire experiment merely to dispel the notion that any top heaviness would invariably result in an alignment with the direction of gravity. The settling velocity *U* of *C. elegans* is ~ 0.5 mm/s (Fig. 4); the animal’s length *L* ~ 1 mm, and the kinematic viscosity of water *ν* ~ 10^−6^ m^2^/s, resulting in *Re* ~ 0.5, which may be enough to counter the tendency to rotate due to small top heaviness, if any.

In conclusion, the absence of gravitaxis in paralyzed animals and an assortment of mutants suggests that *C. elegans* is either not top heavy or, if it is, top heaviness does not cause gravitaxis.

### Gravitaxis is not caused by hydrodynamic effects

*C. elegans* swimming is reasonably well-understood, has been extensively studied experimentally, and reproduced in numerical simulations [21]. The animal swims by propagating undulatory waves from head to tail in the dorsal-ventral plane. This motion is induced by alternating contraction and relaxation of dorsal and ventral muscle groups located along the swimmer’s body [22]. Could the animal’s gait cause it to align with the direction of gravity? If the animal were unable to sense the direction of gravity, one would expect that the pitch angle of its plane of dorsoventral body undulations would be randomly oriented in space and that the animal would not have any preference to swim in the direction of the gravity vector. This is clearly not the case.

Suppose that the animal is top heavy, could swimming cause it to orient with the direction of gravity while such alignment would not take place in the absence of swimming? Although we are not aware of any mechanism that would facilitate such a feat, we tested this hypothesis by examining the animal’s propensity to gravitax as a function of its motility (Fig. 6A). We quantify animal swimming vigor with the Womersley number $Wo=A\sqrt{2\pi f/\nu }$, where *A* is the gait amplitude and *f* the gait frequency. Figure 6A depicts the concentration parameter (*λ*) as a function of *Wo* at the greatest depth (residence time) for which we have experimental data. Figure 6B depicts *λ* as a function of depth. We find no correlation between gravitaxis and motility. Mutant worms that are either less motile or more motile than wild-type worms fail to gravitax. Among animals with the same motility, some gravitax while others do not. For example, the swimming gaits of the gravitaxis-competent WT and the gravitaxis-incompetent *osm-6* are similar, with approximately the same frequency, the same amplitude, and the same *Wo* number (Fig. S24). In summary, variations in swimming gaits of gravitaxis-competent and gravitaxis-incompetent animals do not suggest that the swimming gait of certain animals would cause them to swim downward and that of others would not.

Could the interaction between the flow field induced by the animal’s swimming and the flow field resulting from the animal’s downward sedimentation rotate the animal to align it with the gravity vector? If such a mechanism existed, symmetry considerations would suggest that animals suspended in a liquid denser than themselves (settling upwards) would align in the upward direction. Our experiments using the LUDOX solution that is slightly denser than the animals (Fig. 5) indicate that this is not the case. Wild-type animals turn downwards regardless of whether they are suspended in a lighter or heavier medium than themselves. Hence, hydrodynamic interactions are an unlikely cause of gravitaxis.

Our findings indicate that downward swimming is caused neither by animal’s mass distribution nor by hydrodynamics and therefore is most likely mediated by the animal’s nervous system. Could the observed downward swimming behavior be caused by the nervous system sensing physical factors other than gravity?

### Downward swimming behavior is unlikely to be caused by a sensory input other than gravity

Our experiments took place in a vessel at equilibrium with the ambient and subjected to uniform room light and temperature. In the absence of gradients of light intensity or temperature, phototaxis or thermotaxis is unlikely. While *C. elegans* prefers low oxygen tensions [23, 24], our observations are unlikely to be explained by aerotaxis behavior because the concentrations of gases are nearly uniform in our aqueous column, aside from O_2_ consumption and C0_2_ release by the worms themselves, which is likely negligible on the time scale of our experiments. Finally, by experimenting with the Australia-derived wild-type isolate AB1 and with well-fed and starved worms, we largely exclude the possibility that gravitaxis is explained by the previously described magnetotaxis behavior [20].

Although we cannot exclude with absolute certainty that yet-unappreciated factors other than gravity affect the behavior of the animals in our experiments, our collective observations indicate with a high likelihood that the animals respond to gravity.

If neural circuits are responsible for sensing and responding to gravity, we should be able to render the animal gravitaxis-incompetent by switching off one or more genes associated with such neuronal circuits. Here, we do so by experimenting with mutant animals that are defective in various neural functions.

### Gravitaxis requires ciliated neurons and dopaminergic neurotransmission

Many sensory functions of *C. elegans* such as olfaction, gustation, thermosensation, nose-touch, and electrosensation are mediated by neurons that extend cilia to the nose of the animal. Animals mutant for the genes *osm-6* or *che-2*, in which sensory cilia are globally disrupted [9], did not gravitax. In contrast, animal mutants in the genes *mec-4* and *mec-3*, which are involved in the transduction of mechanical stimuli to the body, did gravitax. These observations suggest that the neural mechanism of gravitaxis requires the function of one or more sensory ciliated neurons.

To begin identifying the relevant neurotransmitter systems, we examined the role of dopamine neurotransmission. Dopamine is required for the function of certain mechanosensory behaviors in *C. elegans*, including the sensation of bacterial particles [12] and habituation to mechanosensory stimulation to the body [25, 26]. In hermaphrodites, there are eight dopaminergic neurons—four CEPs in the nose, two ADEs in the head, and two PDEs in the body [27]—that signal via extra synaptic mechanisms to modulate mechanosensory neuron responses to body touch [25], chemical responses of the nociceptive ASH neurons [28], and motor neuron function [29]. All dopaminergic neurons are ciliated with putative mechanosensory dendrites; the CEPs have been shown to respond directly to mechanical stimuli [30, 31].

Our experiments with mutants harboring a defective dopamine biosynthetic enzyme indicate that dopamine is required for gravitaxis. Our observations of defective gravitaxis in two *cat-2* alleles and of pharmacological restoration of *cat-2* gravitaxis by exogenous dopamine demonstrate an essential role for dopamine in this behavior.

Dopamine could be actively required at the time of liquid suspension for the animal to successfully gravitax. Alternatively, dopamine could be required for the development of the competency for gravitaxis. Our experiments do not clearly distinguish between these two roles. Gravitaxis behavior in *cat-2* mutants was restored by cultivation during larval development with exogenous dopamine. In contrast, gravitaxis behavior was not restored by the presence of exogenous dopamine during only the time of the assay. While these observations might suggest a developmental role for dopamine, the observation of strong rescue of gravitaxis when we combined exposure to dopamine larval development with exposure to dopamine during the experiment suggests at least some active role for dopamine during gravitaxis behavior.

In *C. elegans*, dopamine activates the mammalian D1-like dopamine receptors DOP-1 and DOP-4 and the mammalian D2-like receptors DOP-2 and DOP-3 [32]. DOP-1 and DOP-3 act antagonistically in motor neurons [29]. Our experiments suggest that DOP-1, DOP-2, and DOP-3 are all required for gravitaxis behavior. Since these dopamine receptors are expressed widely in the nervous system [33], an important future direction in dissecting the gravitaxis circuit will be to determine where these dopamine receptors are acting.

### Hypothesis: the animal’s head is the test mass (statolith) for gravity sensing

The organ responsible for gravity sensing in *C. elegans* is elusive. A few invertebrates use their head as the test mass (statolith) to detect the direction of gravity [2]. We do not have proof that the worm’s head serves as the statolith. However, when on agar, the animal’s head is supported by the agarose matrix, is not free to “fall,” and cannot serve as a test mass. Although speculative, the absence of gravitaxis while on agar is consistent with the notion that the animal’s head serves as a test mass.

If the head does, indeed, serve as the test mass, how is information on head position transmitted to the nervous system? The RIA interneurons encode head orientation [34] and could provide input to gravity sensing circuits. Interestingly, at least one dopamine receptor, DOP-2, is expressed in RIA [35]. Future experiments could test the role of RIA in gravitaxis by genetically ablating it.

The ethological significance of gravitactic behavior in *C. elegans*, if any, is not known*.* Gravitaxis may be an ancestral trait. *C. elegans* has other traits such as electrotaxis—the role of which in the animals’ natural ecology is also unknown.

### *C. elegans* as a discovery platform for finding genes required for gravity perception

Our method of monitoring the swimming orientation of downward sedimenting animals provides a means to identify genes required for gravity perception. In combination with the rich library of *C. elegans* mutants and genetic tools to silence or activate neurons, this assay could identify the molecular pathways required for gravity sensing and behavioral response.

Our experimental assay in which we suspended animals in a medium slightly denser than the animals could potentially serve as a high-throughput, forward genetic screening platform to sort out active animals that are defective in gravitaxis and identify the genes that are required for gravitaxis. Following mutagenesis, active animals can be suspended in a solution slightly denser than themselves, gravitaxis-competent animals would swim downward while gravitaxis-defective animals would float to the top. The latter can be collected and subjected to molecular genetic studies. We have previously used a somewhat similar approach to identify genes responsible to sleepiness [36].

Such assays can be used to decipher the neural circuits responsible for gravity sensation and identify which sensory neurons encode gravitational stimuli, which ones decode this information, and what molecular pathways facilitate signaling. While there are significant anatomical differences among animals, there is a remarkable conservation across phylogeny at the molecular level. Knowledge gained in such studies may be beneficial to human health.

## Conclusions

Sensing the direction of the Earth’s gravitational field is essential for the spatial orientation and navigation of animals, including humans. The molecular and circuit mechanisms of gravity sensing and responding to gravity are elusive. We have shown that *C. elegans* responds to gravity and that specific neural circuits are required for responding to gravitational clues. Our work suggests the possibility of leveraging the powerful genetic and physiological toolkit of *C. elegans* to elucidate the molecular and circuit mechanisms of gravity sensing.

## Materials and methods

### Worm preparation

On the day prior to the experiment, well-fed fourth larval stage hermaphrodites were placed on an agar plate containing a bacterial lawn of OP50. Day 1 adult worms were harvested from the agar plate by floating the worms in M9 buffer and then transferring the worm suspension into a 1.5-mL conical tube. Following centrifugation (6000 rpm, 2000 rcf, LAB-PC100 Mini centrifuge) for a few seconds to sediment the worms, the supernatant was decanted. The worms were then washed three times with 1 mL M9 buffer by repeating the centrifugation/decanting steps. We experimented mostly with “recently-fed” worms—the time elapsed from floating the worms off their cultivation plate to the completion of the experiment was < 30 min. A few of the experiments were intentionally carried out with “starved” worms—the time elapsed from floating the worms off their cultivation plate to the start of the experiment was ~ 1 h.

To paralyze wild-type worms by heat shock, we suspended the worms in 1 mL M9 buffer in a 1.5-mL conical tube and placed them for 1 h in a water bath at 40 °C. The experiment was then performed at room temperature (21~22 °C) within 30 min from the removal of the animals from the water bath. Observations of these worms showed that they were fully paralyzed for over 30 min after the heat shock.

To transiently paralyze wild-type worms chemically, we suspended the worms in 10 mM sodium azide in M9 buffer for 5 min. We then performed our experiments with the worms suspended in the same 10 mM sodium azide solution.

### Strains

*C. elegans* strains used in our study were N2 (*wild-type*, WT), AB1 (wild-isolate from Australia), CB190 (*unc-54(e190)*), CB1072 (*unc-29(e1072)*), CB1033 (*che-2(e1033)*), PR811 (*osm-6(p811)*), CB1138 *mec-3(e1338)*, TU253 *mec-4(u253)*, CB1112 *cat-2(e1112)*, MT15620 *cat-2(n4547)*, LX636 *dop-1(vs101)*, LX702 *dop-2(vs105)*, and LX703 *dop-3(vs106)*. The strains were obtained from the Caenorhabditis Genetics Center (CGC). Worms were cultivated in a 20 °C incubator on the surface of NGM agar plate (5.5 cm diameter; 11 mL total volume) seeded with *Escherichia coli OP50* as the food source. All experiments were performed with hermaphrodites.

### High-density buffer

To achieve a buffer density greater than that of the worms, we mixed a suspension of colloidal silica particles in water (LUDOX HS-40, Sigma, density ~ 12 nm diameter; 1.3 g/mL at 25 °C [37]) with M9 buffer at a volume ratio 1:2 to form a homogeneous solution with density of 1.1 g/mL, which is slightly greater than the worm’s density (~ 1.07 g/mL [38]). The mixture density was measured directly by weighing 1 mL of solution. At the density used in our experiments, the suspension behaves like a Newtonian homogeneous liquid with a viscosity approximately 7 times that of water [39].

### Dopamine repletion experiment

Animals were exposed to dopamine (DA) by cultivating them on DA-doped agar and/or by adding dopamine to the M9 solution in the cuvette during the experiment. Agar doping: 400 μl of 50 mM DA hydrochloride (Sigma) dissolved in M9 buffer was added to an agar plate seeded with bacteria. Plates were dried for 1 h [13]. We cultivated animals on the treated agar plates from the first larval L1 stage to the young adult stage. Liquid doping: The solution in the cuvette was blended with dopamine to form 50 mM DA hydrochloride solution.

### Experimental apparatus

Cuboid polystyrene cuvettes with a square cross-section 12 mm × 12 mm and heights ranging from 45 to 200 mm filled with M9 buffer at room temperature (21~22 °C) were used in our settling experiments. To verify that the cuvette material does not affect animal behavior, experiments were repeated in glass tubes with similar results. Twenty microliters of a worm suspension at a concentration of about 1.5 worms per microliter was extracted with a plastic tip pipette and transferred into the cuvette by slowly expelling the worms into the cuvette solution either above or below the liquid surface.

### Imaging

The worms were monitored with two cameras (IMAGING SOURCE DMK 33GP031 with a 25-mm lens and IMAGING SOURCE DMK 22BUC03 with a 12-mm lens) acquiring images at 30 frames per second from two orthogonal planes (Fig. 1). One camera focused on the *X*-*Z* plane and the other on the *Y*-*Z* plane at the cuvette’s center. Each image size was 640 × 480 pixels, which results in an aspect ratio of 4:3. As it settled, a worm stayed within the field of view of the two cameras for about 10 s. Images were processed with a Matlab R2018b graphical user interface (GUI), following the scheme described in [40] and outlined below (and in Fig. S1). In the experiments with non-paralyzed worms, only active (swimming) animals were analyzed. The fraction of non-motile worms in these experiments (Table S2) was typically less than 5% with the exceptions of *unc-29* (17%). When studying *unc-54*, we were interested only in fully paralyzed worms (about 33% of the population) and we censored animals that showed any feeble swimming movements.

### Experiments on vertical agar

Two percent agarose (usb, agar, Bacteriological Ultrapure, Type A) was dissolved in a NGM solution and poured into a round dish plate (diameter = 3.5 cm) marked with a cross on its bottom to demarcate four quadrants. After drying the agar, bacteria (*E. coli* OP50) suspension was added to the whole surface of the agar and dried. Adult worms were harvested from their cultivation agar plates, washed with NGM buffer three times, and pipetted as a suspension onto the agar plate. After removing excess liquid, the number of worms in each quadrant of the plate was counted. Then, the agar plate was positioned in the vertical plane with one of the cross legs parallel to the direction of gravity and monitored with a camera. Images were collected at the rate of one image every 20 min for 12 h. At the end of the experiment, the agar plate was inserted on the stage of a stereo microscope and the number of worms in each quarter was counted within less than a minute.

### Image processing

Images were processed with a Matlab R2018b graphical user interface (GUI), following the image processing scheme described in [40] and outlined below.

### Data analysis (Fig. S1)

We denote the coordinates (Fig. 1) in the *X*-*Z* plane as (*x*_*nXZ*_, *z*_*nXY*_) and in the *Y***-***Z* plane as (*y*_*nZY*_, *z*_*nZY*_), where *n* is the frame number. The cameras’ pixel size was correlated with the physical length by placing a ruler in front of the camera at the same distance from the camera lens as the cuvette’s mid-plane. The pixel size *P* in the *X-Z* plane was ${P}_{XZ}=18.8\frac{\upmu \mathrm{m}}{\mathrm{pixel}},$ and in the *Y* plane was ${P}_{YZ}=22.0\frac{\upmu \mathrm{m}}{\mathrm{pixel}}.$ Accordingly, the distances *Rx*, *Ry*, and *Rz* between the animal’s head (*H*) and tail (*T*) along the *x*, *y*, and *z* coordinates are, respectively, ${R}_{n,x}^{TH}={P}_{XZ}\left({x}_{n, XZ}^T-{x}_{n, XZ}^H\right)$; ${R}_{n,y}^{TH}={P}_{XZ}\left({y}_{n, XZ}^T-{y}_{n, XZ}^H\right)={P}_{YZ}\left({y}_{n, YZ}^T-{y}_{n, YZ}^H\right)$; and ${R}_{n,z}^{TH}={P}_{YZ}\left({z}_{n, YZ}^T-{z}_{n, YZ}^H\right)$.

The distance between the animal’s head and tail

4 $$ \left\Vert \overline{TH_n}\right\Vert =\sqrt{{R_{n,x}^{TH}}^2+{R_{n,y}^{TH}}^2+{R_{n,z}^{TH}}^2} $$

The inclination (polar) angle with respect to the vertical axis

5 $$ {\theta}_n={\cos}^{-1}\frac{R_{n,z}^{TH}}{\left\Vert \overline{TH_n}\right\Vert }. $$

The angle *θ =* 0° corresponds to the upward direction. The azimuthal angle

6 $$ {\varphi}_n={\cos}^{-1}\frac{R_{n,x}^{TH}}{\sqrt{{R_{n,x}^{TH}}^2+{R_{n,y}^{TH}}^2}}. $$

The algorithm was verified by reproducing the dimensions and inclination angle of a 3D-printed calibration jig (Fig. S2).

To monitor the worms’ orientation as a function of residence time in solution, images of worms were recorded and processed at various positions beneath the liquid surface (Fig. 1). The fields of view of the cameras covered 640 × 480 pixels, which corresponds to a vertical distance of approximately 9 mm.

The position of the worm’s centroid (geometric center)

$$ {x}_{n, XZ}^C=\frac{\left({x}_{n, XZ}^H+{x}_{n, XZ}^T\right)}{2};{y}_{n, XZ}^C=\frac{\left({y}_{n, XZ}^H+{y}_{n, XZ}^T\right)}{2}=\frac{\left({y}_{n, YZ}^H+{y}_{n, YZ}^T\right)}{2};\mathrm{and}\kern0.24em {z}_{n, YZ}^C=\frac{\left({z}_{n, YZ}^H+{z}_{n, YZ}^T\right)}{2}. $$

The components of the worm’s centroid displacement between subsequent frames are given by $\Delta  {R}_{n,x}^C={P}_{XZ}\left({x}_{n+1, XZ}^C-{x}_{n, XZ}^C\right)$; $\Delta  {R}_{n,z}^C={P}_{XZ}\left({y}_{n+1, XZ}^C-{y}_{n, XZ}^C\right)={P}_{YZ}\left({y}_{n+1, YZ}^C-{y}_{n, YZ}^C\right);$ and $\Delta  {R}_{n,y}^C={P}_{ZY}\left({z}_{n+1, YZ}^C-{z}_{n, YZ}^C\right)$. The displacement of the worm’s centroid between frames

7 $$ \Delta  {D}_n=\sqrt{\Delta  {R_{n,x}^{C^2}}+\Delta  {R_{n,y}^{C^2}}+\Delta  {{\mathrm{R}}_{n,z}^{C^2}}}. $$

The velocity of the worm is

8 $$ {U}_n=\Delta  {D}_n\times 30\ \left(\upmu \mathrm{m}/\mathrm{s}\right), $$

where the factor 30 is the video frame rate. The polar angular velocity

9 $$ {\omega}_n=\left\langle {\theta}_{n+1}-{\theta}_n\right\rangle \times 30\;\left(\mathrm{rad}/\mathrm{s}\right). $$

### Image processing algorithm

Images were processed with Matlab R2018b, following the image processing scheme described in [40]. Briefly,

1. The grayscale threshold for detecting worms was manually adjusted using the ImageJ program to transform the captured grayscale images into a binary scale: zero for black (outside the worm) and one for white (inside the worm). The grayscale ranges from 0 to 255. The threshold for detecting a worm varied among experiments and ranged from 32 to 85.
2. Background subtraction. In the first frame (*n* = 0) of each video, we manually defined an imaging region. Pixels in the region outside this imaging region were assigned a zero (black) value. The most frequently occurring binary value (mode) within our imaging region, excluding the worm, was subtracted from all subsequent frames in the video.
3. A tight bounding rectangle, containing the worm, was manually defined in the first frame. The positions of the tips of worm’s head and tail were manually marked. We then defined in frame (*n* + 1) an extended bounding rectangle with 10 pixels added to the width and length of the bounding rectangle of frame (*n*) to form a search-region for the worm following its displacement in the time span between frame *n* and frame (*n* + 1).
4. To smooth the noise, we replaced each pixel’s grayscale value with the average of itself and 8 neighbors in the 3 pixels × 3 pixels surrounding the square.
5. Next, we used Matlab’s edge detection function CANNY to locate the worm’s boundary. The contour was smoothed with Matlab’s functions “strel,” “imdilate,” “imerode,” and “imfill.”
6. The worm’s skeleton is defined as the center line of the worm’s body and is found by thinning the worm’s body from both sides simultaneously. The end points of this skeleton were defined as the worm’s head and tail. The head was distinguished from the tail manually in the first image. The angle formed by the line connecting the head and the tail relative to the upward direction was defined as the angle of inclination *θ* (Fig. 1).
7. Once the positions of the end points were determined in the two focal planes, 3D coordinates were computed for the head and tail positions.
8. Immobile (non-swimming) worms were excluded from analysis except for experiments purposely carried out with paralyzed worms (e.g., heat-killed N2 and sodium azide-treated N2) and motion-impaired worms (e.g., *unc-54*).

While we minimized the possibility of two or more worms crossing each other’s paths by experimenting with a dilute worm suspension (1.5 worms/μL), images in which worm paths overlapped, as assessed by visual inspection, were censored. We estimate that less than 15 frames of all frames analyzed were censored.

### Raw data

The raw data from our experiments is provided in an accompanying excel file (Additional File 2: Raw Data). The excel sheets include the animal strain, place and date of the experiment, and the orientation angle as a function of the position of the animal’s centroid beneath the liquid surface.

### Kernel distribution estimate (KDE)

Statisticians use the kernel distribution estimate (KDE) to estimate the probability distribution function (*pdf*) based on experimental data. To construct the KDE and the cumulative distribution function (CDF) from our data, we used MATLAB^TM^ function “ksdensity.” Figure S3 compares KDEs with histograms for wild-type (A) and paralyzed (B) animals with (black solid line) and without (red solid line) boundary correction. We have used KDE with boundary correction throughout.

To examine the effect of the bandwidth *h* on the KDE, we repeated our calculations with various bandwidths for day 1 adult wild-type animals. Figure S3C demonstrates that the KDE produces similar results for a range of bend widths. Our results therefore are unlikely to be biased by our choice of the bandwidth *h*.

### Directional statistics

To analyze the orientation of the animals, we use spherical coordinates (Fig. S7) with their origin at the animal’s center. Since area elements on the sphere’s surface are non-uniform, it is convenient to model directional distributions on a unit sphere with the von Mises-Fisher (vMF) probability distribution function:

10 $$ f\left(\theta, \varphi \right)=\frac{\lambda }{4\pi Sinh\lambda}{e}^{\lambda \cos \left(\pi -\theta \right)}. $$

In the above, *λ* is the *concentration parameter* (a reciprocal measure of dispersion). *θ* = 0 and *θ* = *π* are, respectively, the upward direction and the downward direction. Since our data suggests (Fig. S5) that the distribution is independent of the azimuthal angle *φ*, we integrated Eq. (10) accounting for the spherical symmetry to obtain:

11 $$ f\left(\theta \right)=\underset{0}{\overset{2\pi }{\int }}f\left(\theta, \varphi \right)\sin \theta d\varphi =\frac{\lambda }{2 Sinh\lambda}{e}^{\lambda \cos \left(\pi -\theta \right)}\sin \left(\pi -\theta \right), $$

where $\underset{0}{\overset{\pi }{\int }}f\left(\theta \right) d\theta =1$. *λ ➔* 0 corresponds to the uniform distribution:

12 $$ \underset{\lambda ->0}{\lim }f\left(\theta \right)=\underset{\lambda ->0}{\lim}\frac{\lambda }{2 Sinh\lambda}{e}^{\lambda \cos \left(\pi -\theta \right)}\sin \left(\pi -\theta \right)=\frac{1}{2}\sin \left(\pi -\theta \right) $$

When *λ = 0*, the *pdf* peaks at *θ*_peak_ = 90° (horizontal direction) and there is an equal probability of finding animals swimming in and against the direction of gravity (as in Fig. S3B). As *λ* increases, so does *θ*_peak_, indicating increasing probability of swimming in the direction of gravity (Fig. S8).

The cumulative distribution function is (*cdf*) (Fig. S9)

13 $$ cdf\left(\theta \right)=\frac{1}{2\mathrm{Sinh}\lambda}\left({e}^{-\lambda \cos \left(\theta \right)}-{e}^{-\lambda}\right). $$

We obtained the experimental estimate of *λ* by fitting equation (S10) to our experimental CDF. We consider cases with *λ* > 1 as exhibiting gravitaxis.

### Statistical (*P*) test

To infer whether the orientational behavior of any strain differs from either that of the wild type (N2) or the paralyzed animals, we carried out the Kolmogorov-Smirnov (KS) test for two samples (MATLAB^TM^ function kstsest2). We tested two null hypotheses to examine whether two samples come from the same distribution. The first null hypothesis ${H}_0^{WT}$ is that any of the probability distributions is like that of the wild type (N2) and the second null hypothesis ${H}_0^{Paralyzed}$ is that any of the distributions is like that of the heat-paralyzed animals at approximately the same depth. The results of these calculations are reported in Table S3 and show that the gravitaxis-capable and gravitaxis-deficient animals follow statistically distinct distributions. Not all gravitaxis-capable animals follow the same distribution as N2.

## Supplementary Information

**Additional file 1:** Supplemental Information. **Figures S1-S30. Tables S1-S3.**

**Additional file 2:** Raw Data.

## Data Availability

All data generated or analyzed during this study are included in this published article and its supplementary information files.
