# Supplementary material for: Caenorhabditis elegans exhibits positive gravitaxis
Source: BMC Biol. 2021 Sep 14;19:186. doi: 10.1186/s12915-021-01119-9 (PMC8439010; doi:10.1186/s12915-021-01119-9)
Supplement: Supplementary file 1 — Additional file 1: Supplemental Information. Figures S1-S30. Tables S1-S3. [file 12915_2021_1119_MOESM1_ESM.pdf]

Additional File 1: Supplemental Information. Figures S1-S30. Tables S1-S3.

Caenorhabditis elegans Exhibits Positive Gravitaxis

Wei-Long Chen[1,2,3], Hungtang Ko[1,4], Han-Sheng Chuang[2], David M. Raizen[3,*], and Haim H. Bau[1,**]

1. Dept. Mechanical Engineering and Applied Mechanics, University of Pennsylvania, Philadelphia, PA. USA
2. Department of Biomedical Engineering, National Cheng Kung University (NCKU), Tainan, Taiwan.
3. Dept. of Neurology, Perelman School of Medicine, University of Pennsylvania, Philadelphia, PA. USA
4. Current address: School of Mechanical Engineering, Georgia Institute of Technology, Atlanta, GA, USA

* raizen@pennmedicine.upenn.edu

** bau@seas.upenn.edu

Table of Contents

S1. Image processing and data analysis
S2. Calibration jig for image processing verification
S3. Kernel distribution estimate (KDE)
S4. Reproducibility and Potential Artifacts
S5. Cuvette material does not affect worms' behavior.
S6. Von Mises – Fisher probability distribution function
S7. Kernel Distribution Estimate (KDE) of well-fed wild type animals at various depths beneath the liquid surface.
S8. Motion-impaired animals do not gravitax
S9. Starved WT and motion-impaired animals (*unc-29*) align with the direction of gravity at a lower rate than well-nourished WT animals.
S10. *C. elegans* mutant for the gene *osm-6* or for the gene *che-2* do not gravitax
S11. *C. elegans* mutant for the gene *mec-3* or for the gene *mec-4* do not gravitax
S12. Dopamine-deficient worms are deficient in gravitaxis.
S13. Pharmacological dopamine rescues gravitaxis defect
S14. Comparison of the swimming gaits of WT, *osm-6*, *cat-2*, and pharmaceutically treated *cat-2*
S15. Well-fed adult worms of the AB1 Adelaide strain align their direction of swimming with the direction of the gravity vector.
S16. *C. elegans* on agar does not gravitax
S17. Top heavy cylinders settle broadside.

Table S1. Summary of our experiments
Table S2. Fraction of censored animals by strain
Table S3. Likelihood test for KDEs

S1. Image Processing and Data Analysis

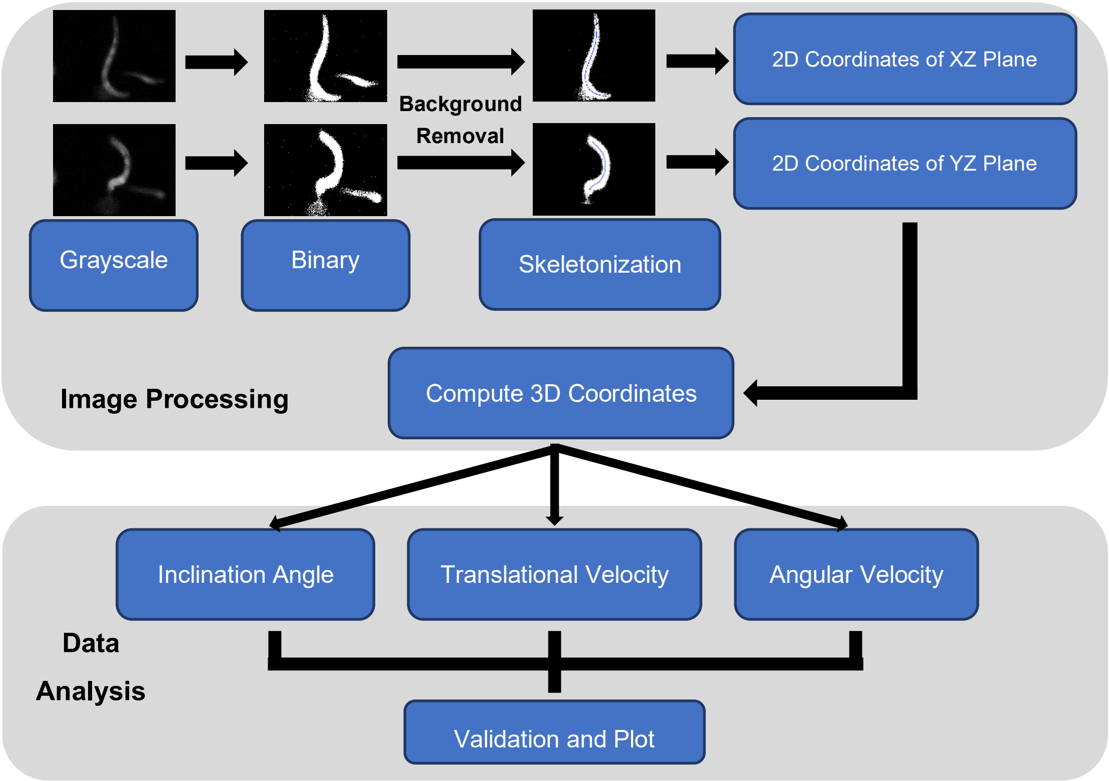

Fig. S1: Schematic depiction of image processing and data analysis. The X-Z and Y-Z projections of a worm were captured with cameras 1 and 2, respectively. The red dot and blue lines in the skeletonization step identify, respectively, the worm's head and skeleton.

S2. Calibration Jig for Image Processing Verification

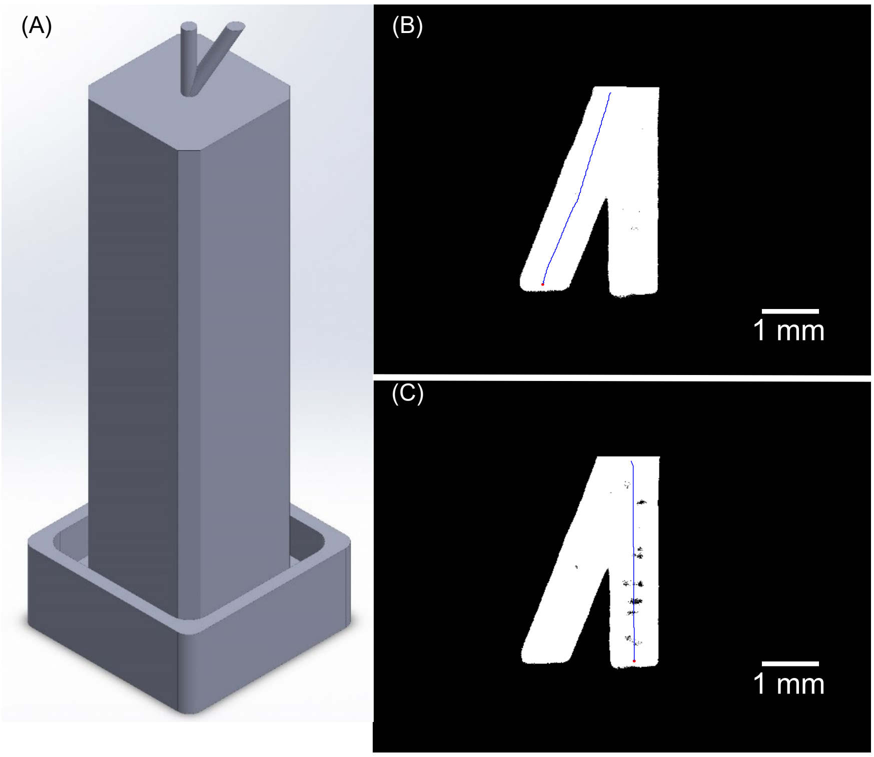

Fig. S2: Calibration jig. (A) The isometric view of the 3D printed calibration jig. Our calculations were verified by reproducing the inclination angle of the jig's legs: $\theta = 150°$ and $\varphi = 45°$ (A) and $\theta = 180°$ (B).

To verify our analysis, we 3D-printed (Formlabs, Form 2) a calibration jig (**Fig. S2**). The jig includes a square base sized to fit tightly into our cuvette, a vertical leg ($\theta = 180°$) located at the jig base's center, and an inclined leg ($\theta = 150°$, $\varphi = 45°$).

We calculated the angle of the inclined leg as θ=149.6°±2.1° and the vertical leg θ=179.81°±1.07°. We estimate that our calculation of the inclination angle is accurate within 3.5°. The cameras are aligned within 7.39 μm in the vertical direction.

S3. Kernel Distribution Estimate (KDE)

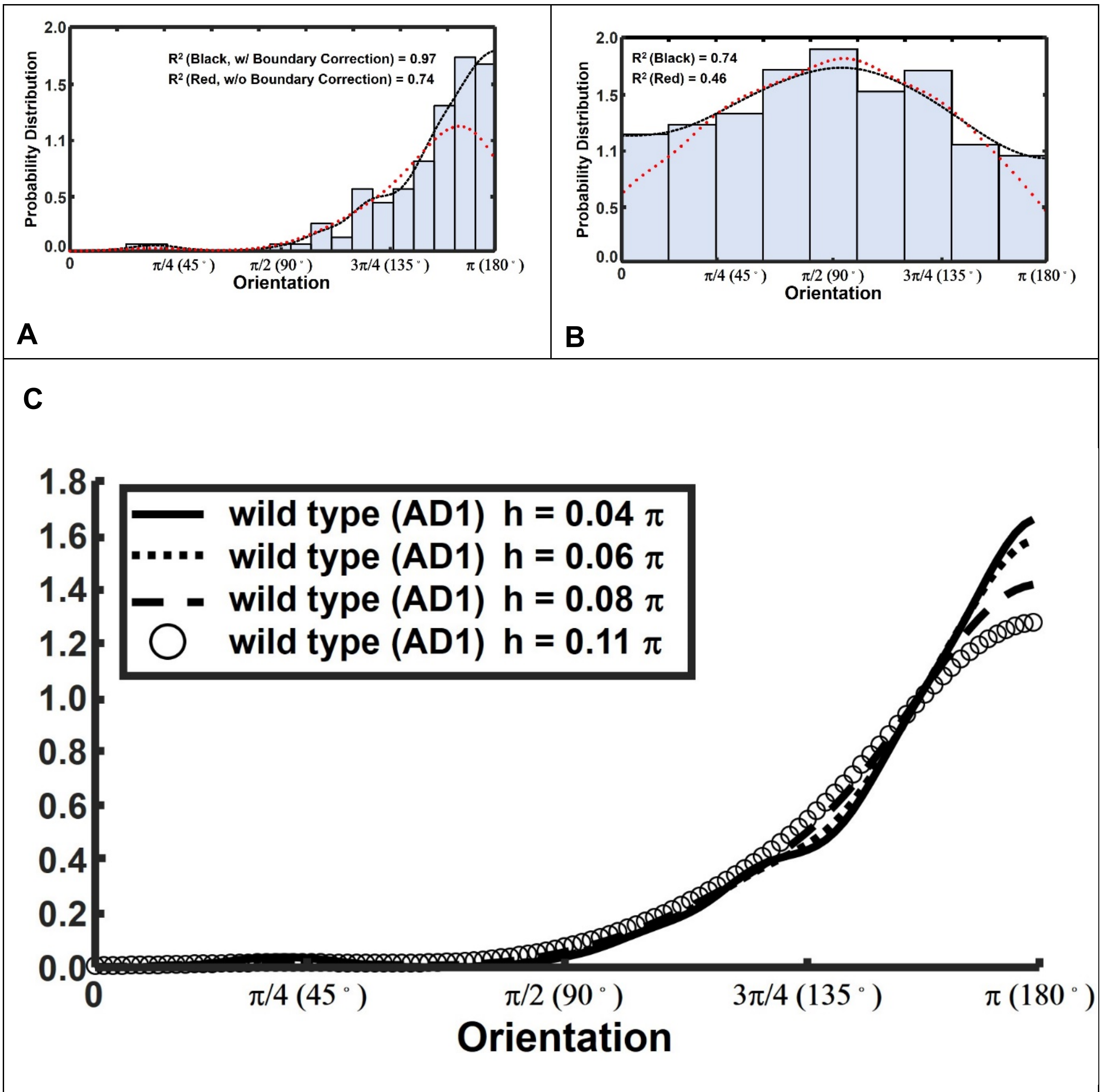

Fig. S3: (**A/B**) Comparison between KDE in the absence (red) and in the presence (black) of boundary correction with the histograms for (**A**) wild type N2 at 200 mm beneath the surface and (**B**) paralyzed WT animals. R^2 is the coefficient of determination whose magnitude depends on the histogram bin's width. The KDE's bandwidth $h=\pi/12$. (**C**) Kernel Distribution Estimate (KDE) with various selections of bandwidth (*h*). Wild type animals in water.

S4. Reproducibility and Potential Artifacts

We repeated our experiments on different days and two different continents with similar results. **Fig. S4** depicts the orientation Kernel Distribution Estimate (KDE) at two different days (left) and two different continents (right) showing similar results.

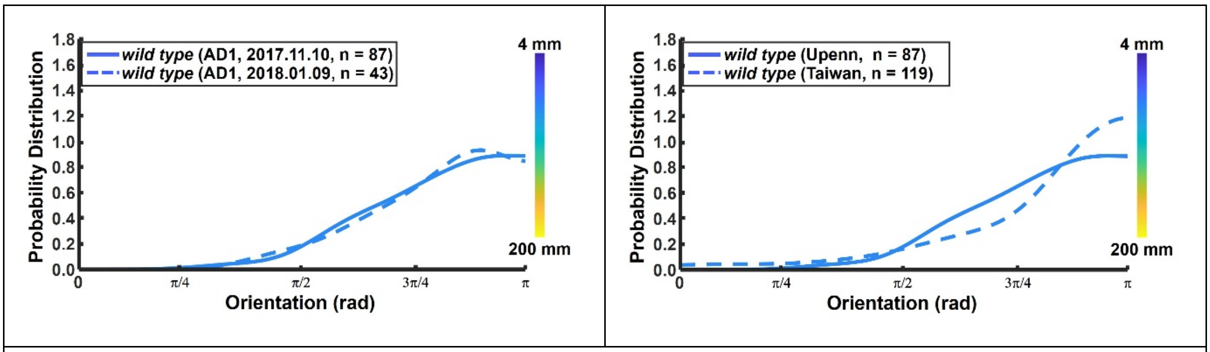

Fig. S4: Polar angle Kernel (probability density) Distribution Estimate (KDE) obtained in two experiments carried out 10 weeks apart (left) and in two different continents (USA and Taiwan).

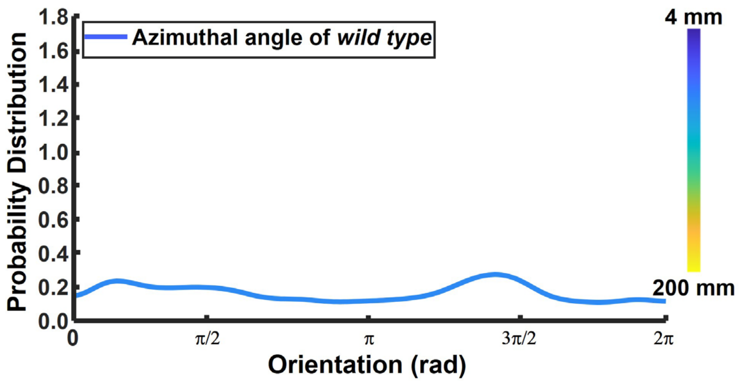

Fig. S5: Kernel Density Estimate (KDE) of the azimuthal angle φ.

To test whether our data may have been biased by convective currents in our experimental apparatus, we examined the Kernel Density Estimate (KDE) of the azimuthal angle φ (**Fig. S5**). Our data shows that worms descended with a nearly uniformly distributed azimuthal angle.

S5. Cuvette material does not affect worms' behavior.

We have carried out most of our experiments with polystyrene cuvettes. On the time scale of our experiments, it is unlikely that the polystyrene would release enough volatile compounds, if any, to affect nematodes' behavior. However, out of the abundance of caution, we repeated our experiments monitoring wild type animals' descent angle in glass tubes (**Fig. S6**), The animals in glass tubes behaved like animals in polystyrene cuvettes.

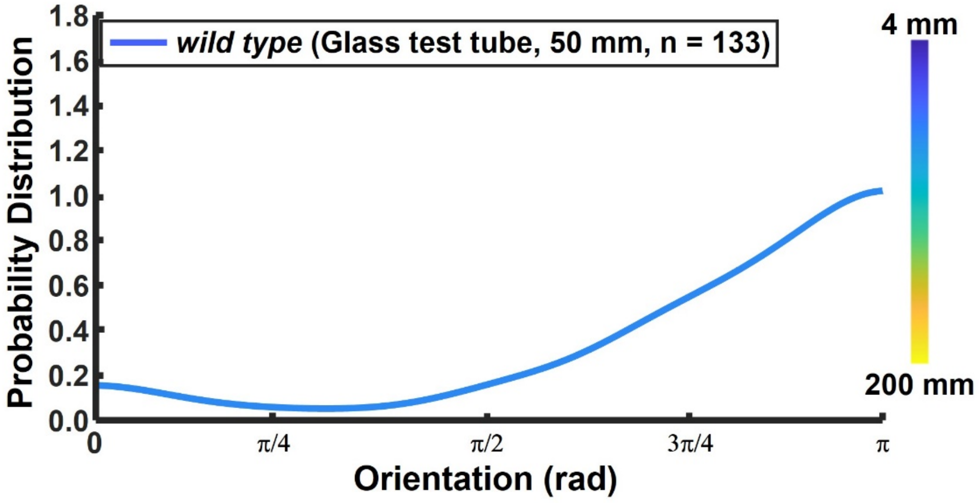

Fig. S6: Kernel Distribution Estimate (KDE) of the orientation angle (θ) at 5 cm beneath the liquid surface of wild type (N2) animals settling in a glass tube. N=133.

S6. Von Mises - Fisher probability distribution function

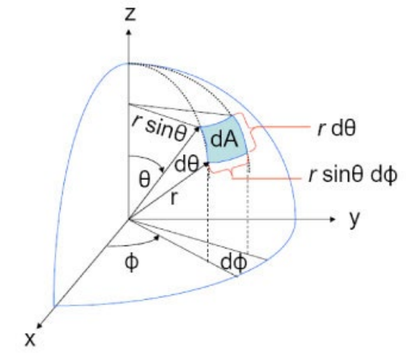

Fig. S7: An area element on a sphere. (https://math.stackexchange.com/questions/131735/surface-element-in-spherical-coordinates)

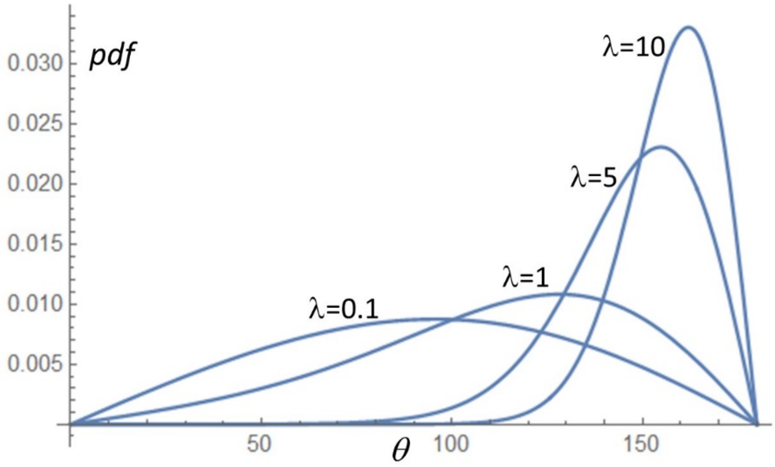

Fig. S8: Von Mises - Fisher probability distribution function(pdf) for various values of the concentration factor. λ = 0.1, 1, 5, and 10.

Fig. S9 depicts the cumulative distribution function (*cdf*) as a function of θ when λ=0.1, 1, 5, and 10.

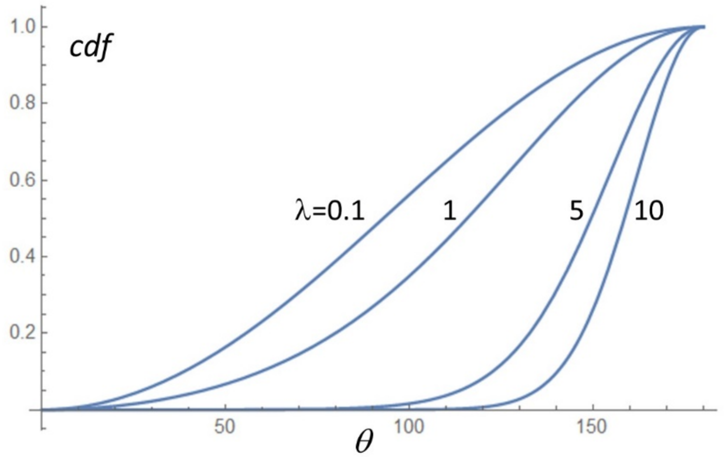

Fig. S9: Von Mises - Fisher cumulative distribution function (*cdf*) for various values of the concentration factor λ = 0.1, 1, 5, and 10.

S7. Kernel Distribution Estimate of well-fed wild-type animals at various depths beneath the liquid surface

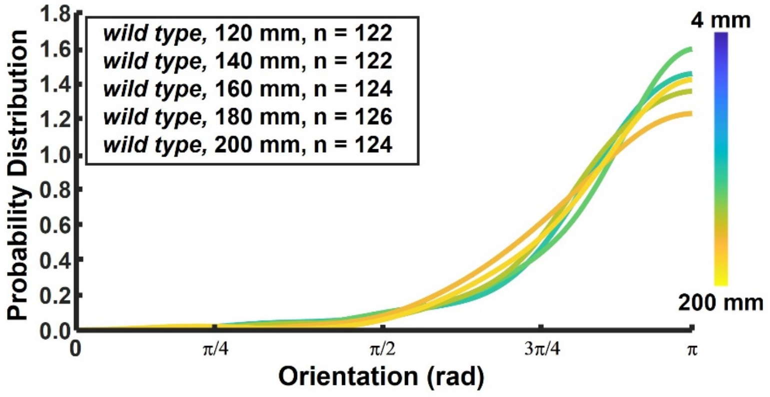

Fig. S10: **Wild-type worms retain their orientation after a certain residence time**. Kernel-density estimate of wild-type swimmers' orientation angle (θ) at positions 120, 140, 160, 180, and 200 mm beneath the liquid surface.

S8. Motion-impaired animals do not gravitax

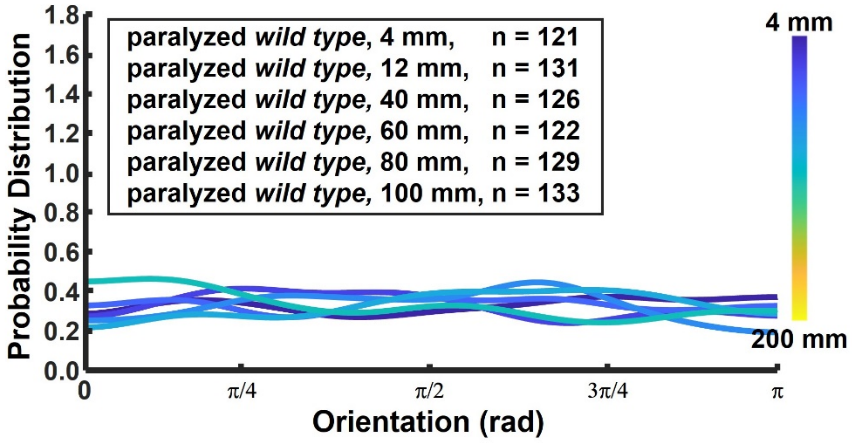

Fig. S11: **Paralyzed WT worms retain random distribution of their orientation as they settle in solution.** Kernel (probability) Density Estimate (KDE) of heat-shock paralyzed WT animals at depths ranging from 4 mm to 100 mm beneath the liquid surface. Similar results were obtained at the depth of 200 mm (not shown). The bandwidth of the KDE smoothing window is 15. This figure augments the data presented in the main text (**Fig. 3** for longer residence times. **Fig. 3** and this figure demonstrate that orientational KDE of paralyzed WT animals does not vary with depth and residence time.

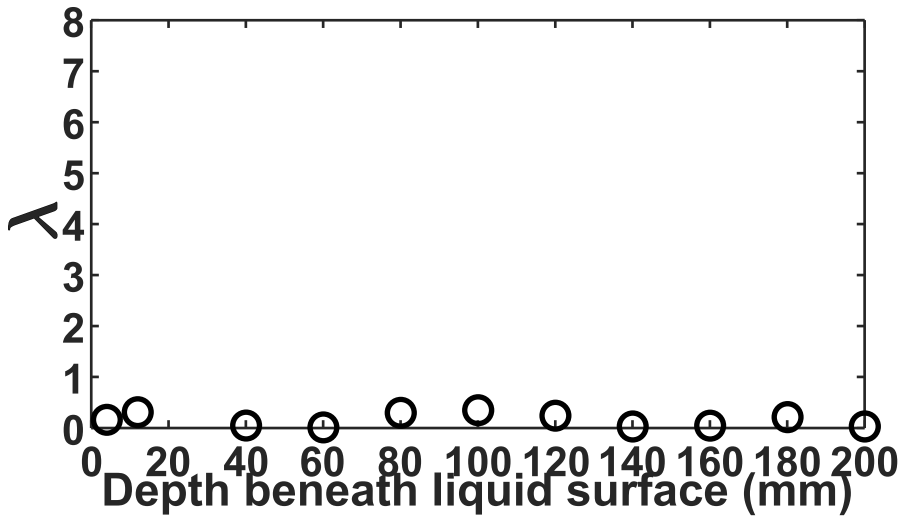

Fig. S12: **Paralyzed (heat-shocked) WT animals: concentration parameter λ as a function of depth (residence time).** λ remains nearly zero, independent of depth (and hence independent of residence time in solution), indicating that the animal's orientation is randomly distributed.

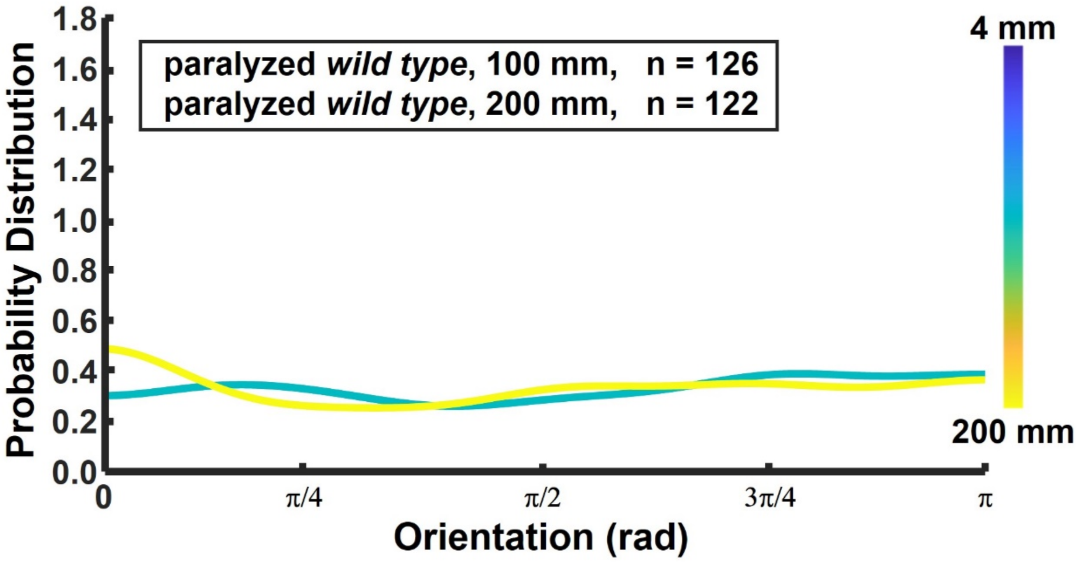

Fig. S13: **Chemically (sodium azide) paralyzed WT worms retain random distribution of their orientation as they settle in solution.** Kernel (probability) Density Estimate (KDE) of chemically - paralyzed WT animals at 100 mm (N=126) and 200 mm (N=122) beneath the liquid surface. The bandwidth of the KDE smoothing window is 15.

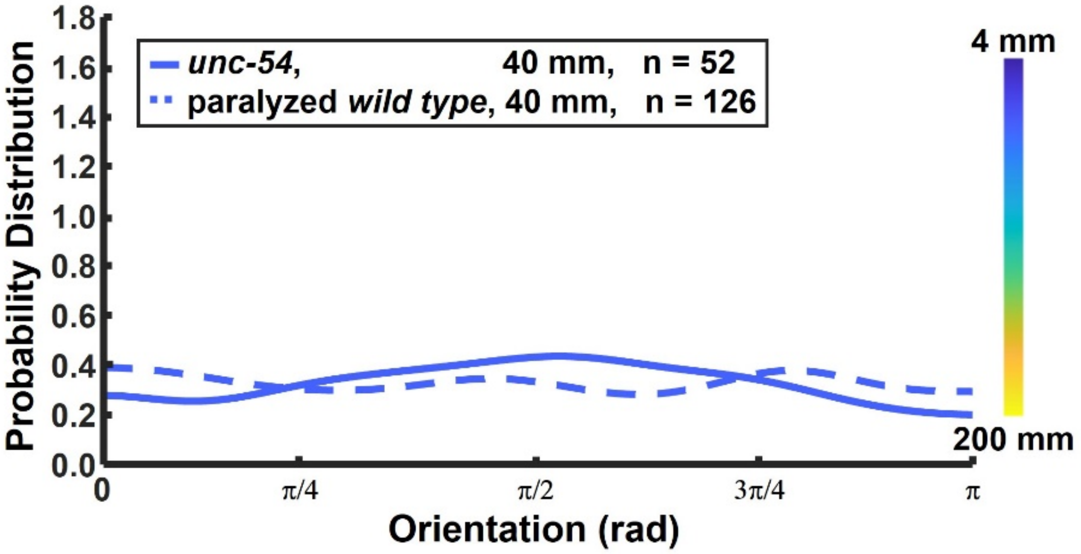

Fig. S14: **KDE of *unc-54* is compared with that of paralyzed (heat-shocked) WT at depth d = 40 mm beneath the water surface.** The two KDEs are similar and consistent with a random distribution in the orientation angle.

S9. Starved WT and motion-impaired animals (*unc-29*) align with the direction of gravity at a lower rate than well-nourished WT animals.

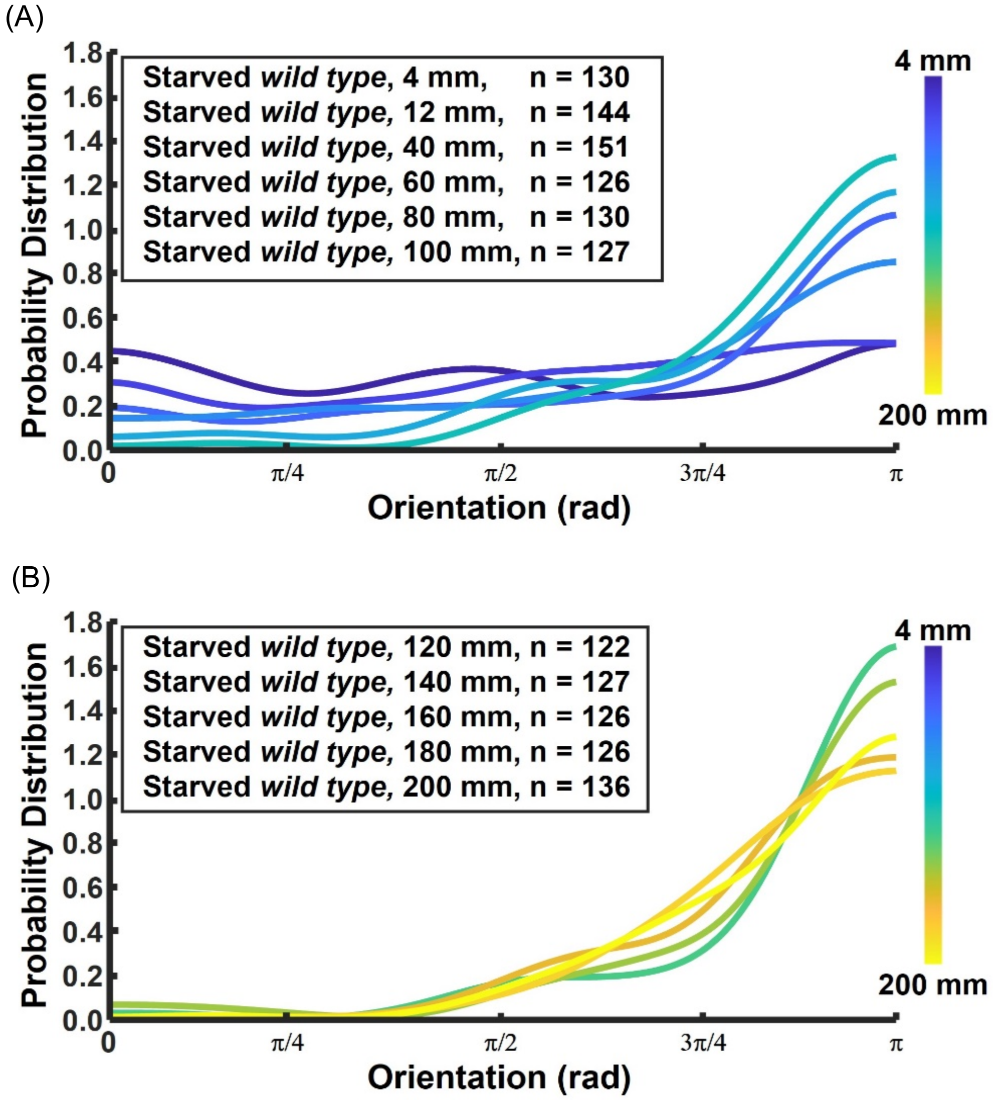

Fig. S15: **Starved (>1 h after last feeding) WT animals align with the direction of the gravity vector.** (**A**) Kernel (probability) density estimate (KDE) of starved WT animals at depths ranging from 4 to 100 mm beneath the liquid surface. The bandwidth of the KDE smoothing window is $\pi/12$. (**B**) Kernel (probability) density estimate (KDE) of starved WT animals at depths ranging from 120 to 200 mm beneath the liquid surface. The bandwidth of the KDE smoothing window is $\pi/12$.

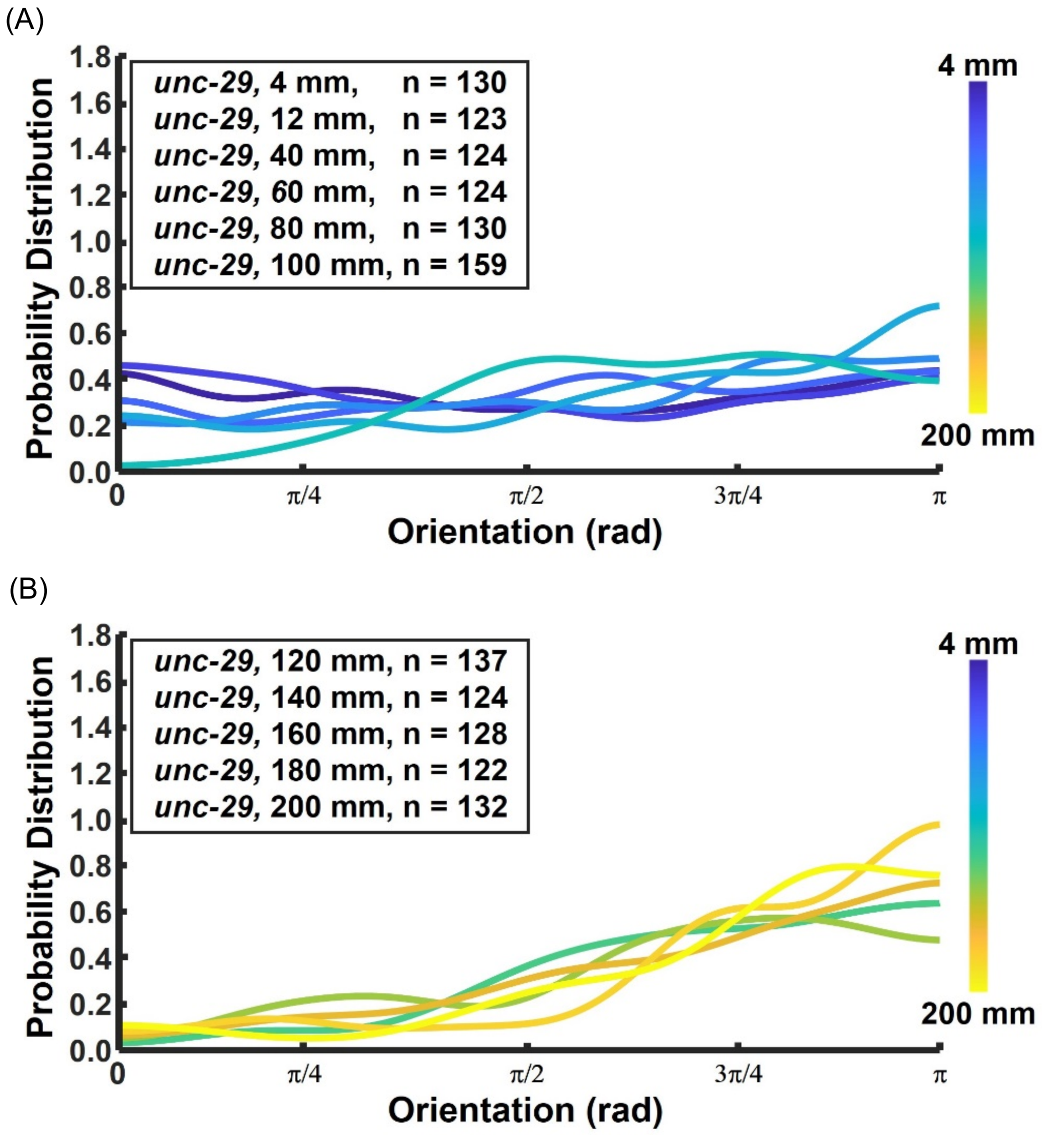

Fig. S16: **Animals defective in neuromuscular junction function** (*unc-29*) **align slowly with the direction of the gravity vector.** (A) Kernel (probability) Density Estimate (KDE) *unc-29* mutants at depths ranging from 4 to 100 mm beneath the liquid surface. The bandwidth of the KDE smoothing window is π/12. (B) Kernel Density Estimate (KDE) *unc-29* mutants at depths ranging from 120 to 200 mm beneath the liquid surface. The bandwidth of the KDE smoothing window is π/12.

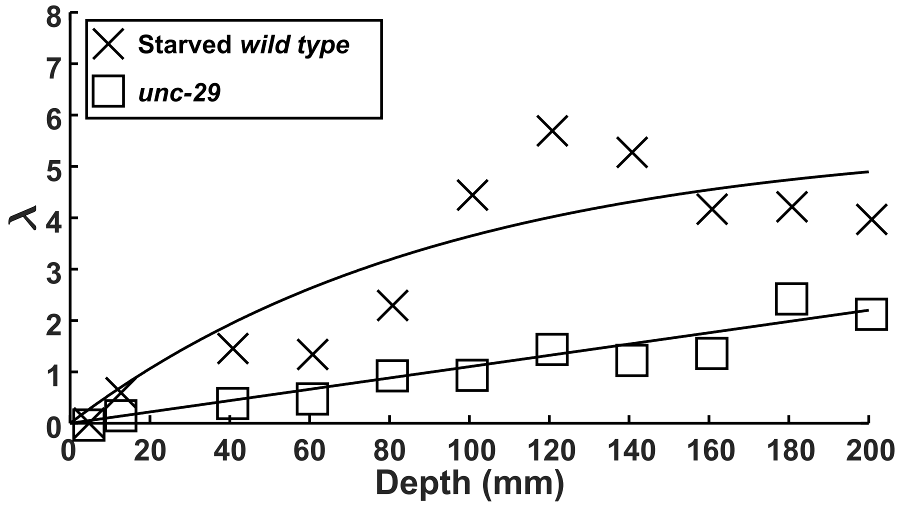

Fig. S17: **The concentration factor of starved WT animals and *unc-29* mutants increases as their submersion depth (residence time) increases.** The increase in the concentration factor indicates alignment with the direction of the gravity vector. The solid lines are best fit curves (equation 3) with λ_∞ ~ *5.56* and β~ *0.011 mm*$^{-1}$ (starved WT) and λ_∞ ~ *12.00* and β~ *0.001 mm*$^{-1}$ (*unc-29*).

S10. *C. elegans* mutant for the genes *osm-6* or *che-2* do not gravitax

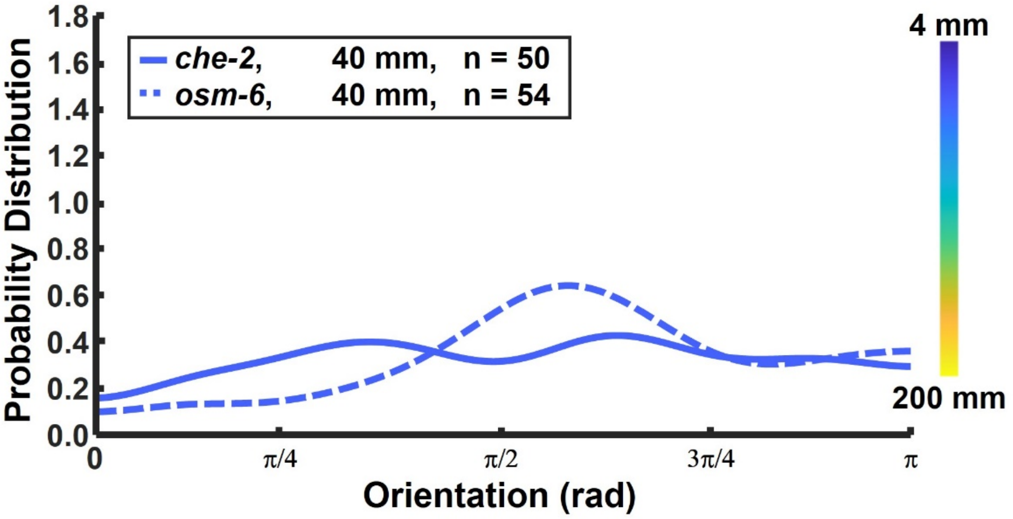

Fig. S18: **Sensory mutants *che-2* and *osm-6* show defects in downward orientation.** Kernel-density estimate plot of angle of descent of sensory mutants at 40 mm beneath liquid surface. The distributions of angles of descent of *che-2* and *osm-6* mutants are all broader than that of wild-type animals and approximate random distribution. Compared to WT distribution, $p \leq 0.0001$ (Mann Whitney Test). $N_{che\text{-}2}$=50, and $N_{osm\text{-}6}$=54. In depicting the KDE curves, we used Matlab^{TM} default values.

S11. *mec-3* and *mec-4* mutants show essentially normal gravitaxis while *che-2* and *osm-6* are defective in gravitaxis.

(A)

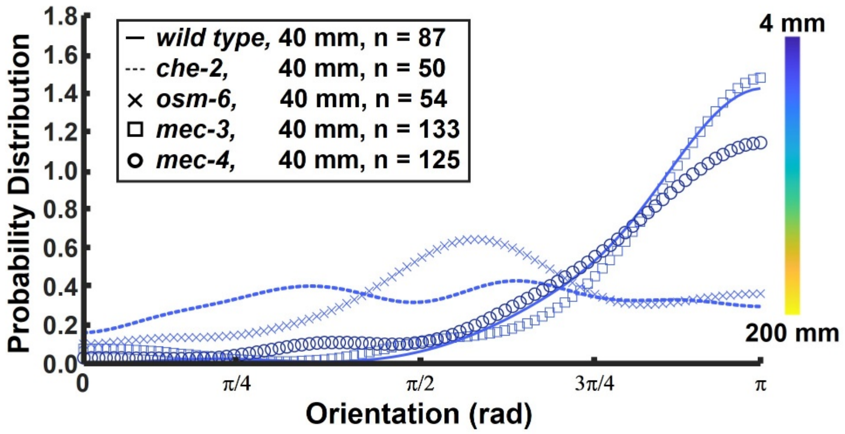

(B)

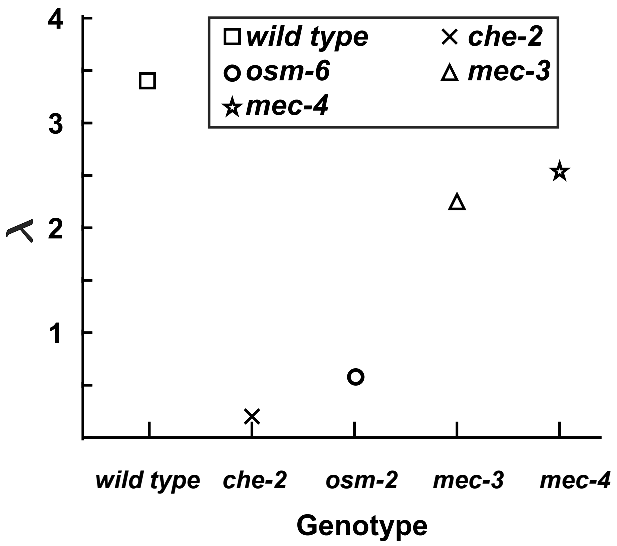

Fig. S19: **Ciliated sensory neuron mutants show defects in downward orientation.** (**A**) Kernel Density Estimate (KDE) plot of angle of descent of sensory mutants and of wild-type controls and (**B**) the concentration factor of the various strains. The distributions of angles of descent of *che-2* and *osm-6* mutants are all broader than that of wild-type animals and show an average close to 90°. The concentration factor and the average angles of descent of the mechanosensory mutants *mec-3* and *mec-4* are lower but not significantly different from that of wild-type animals. N_{WT}=87, N_{che-2}=50, N_{osm-6}=54, N_{mec-3}=133, N_{mec-4}=125. d=40 mm. In depicting the KDE curves, we used Matlab™ default values.

S12. Dopamine-deficient worms are deficient in gravitaxis.

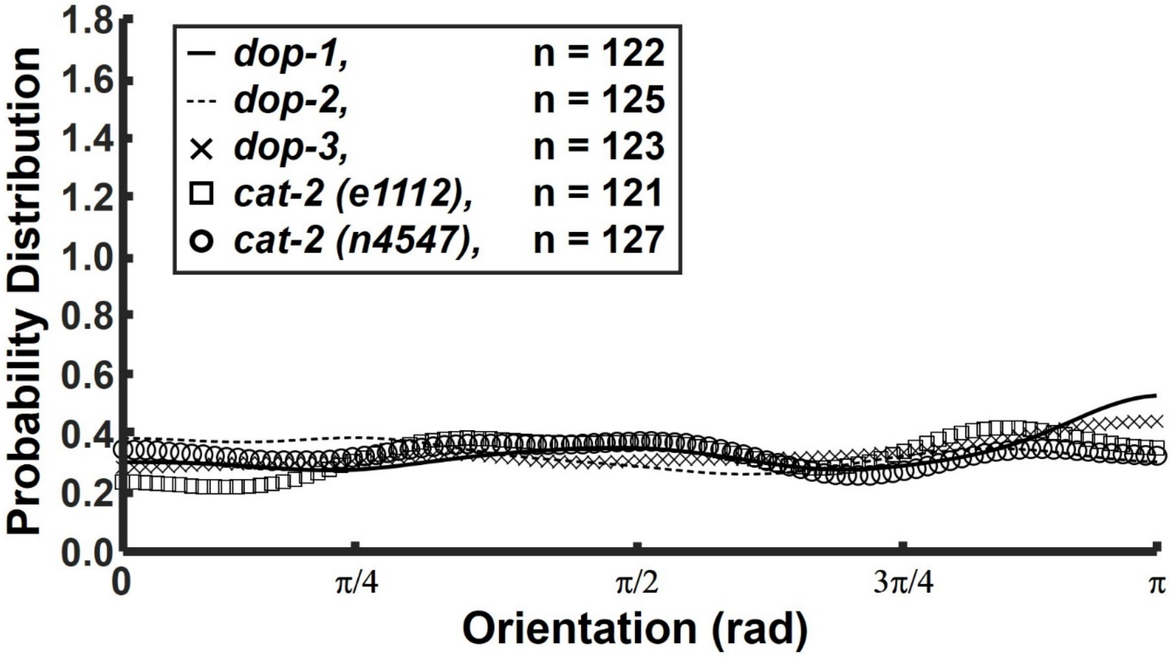

Fig. S20: **Dopamine deficient mutants (*cat-2, dop-1, dop-2, and dop-3*) are deficient in gravitaxis.** Kernel Density Estimate (KDE) plots of the angle of descent of the dopamine deficient mutants: *cat-2* (N=121)*, dop-1* (N=122)*, dop-2* (N=125)*, and dop-3* (N=123) at 200 mm beneath the liquid surface.

S13. Pharmacological dopamine rescues gravitaxis defect in *cat-2*

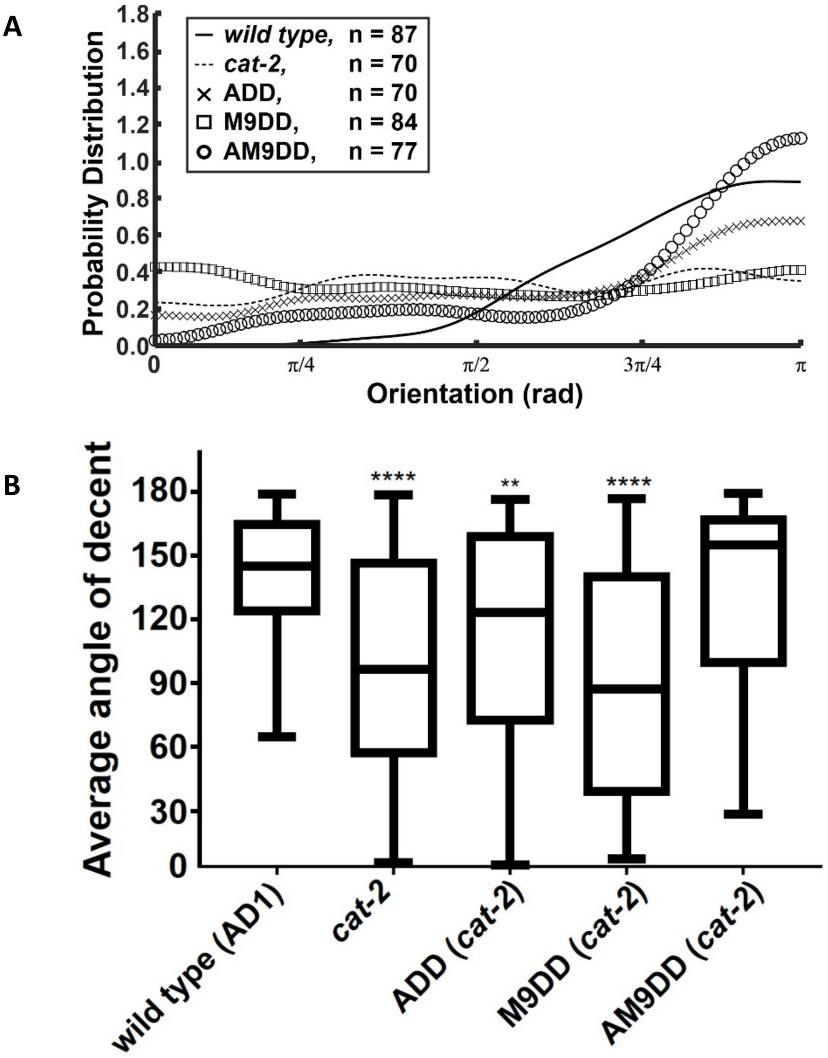

Fig. S21: Dopamine rescues the *cat-2* gravitaxis defect. (A) and (B) show the Kernel-density plot and box plot, respectively. The distributions of angles of descent of *cat-2* untreated mutants, and *cat-2* mutants treated with dopamine in M9 solution are broader than that of wild-type animals and show an average inclination angle close to 90°. The distribution of angles of descent of *cat-2* mutants treated with dopamine both in agar and in M9 are not significantly different from that of wild-type animals ($p = 0.86$), whereas the distribution of angles of descent of *cat-2* mutants treated with dopamine only in the agar was close to but still significantly different from that of wild-type animals ($p \leq 0.01$ **). The distribution of angles of descent of *cat-2* mutants treated with dopamine only in the M9 was not different from untreated *cat-2* mutants and significantly different from that of wild-type animals ($p \leq 0.0001$ ****). Mann Whitney test, $N_{cat\text{-}2}$=70, N_{ADD}=70, N_{M9DD}=84, N_{AM9DD} =77. In depicting the KDE curves, we used Matlab^{TM} default values.

S14. Comparison of the swimming gaits of WT, *osm-6*, *cat-2*, and pharmaceutically treated *cat-2*

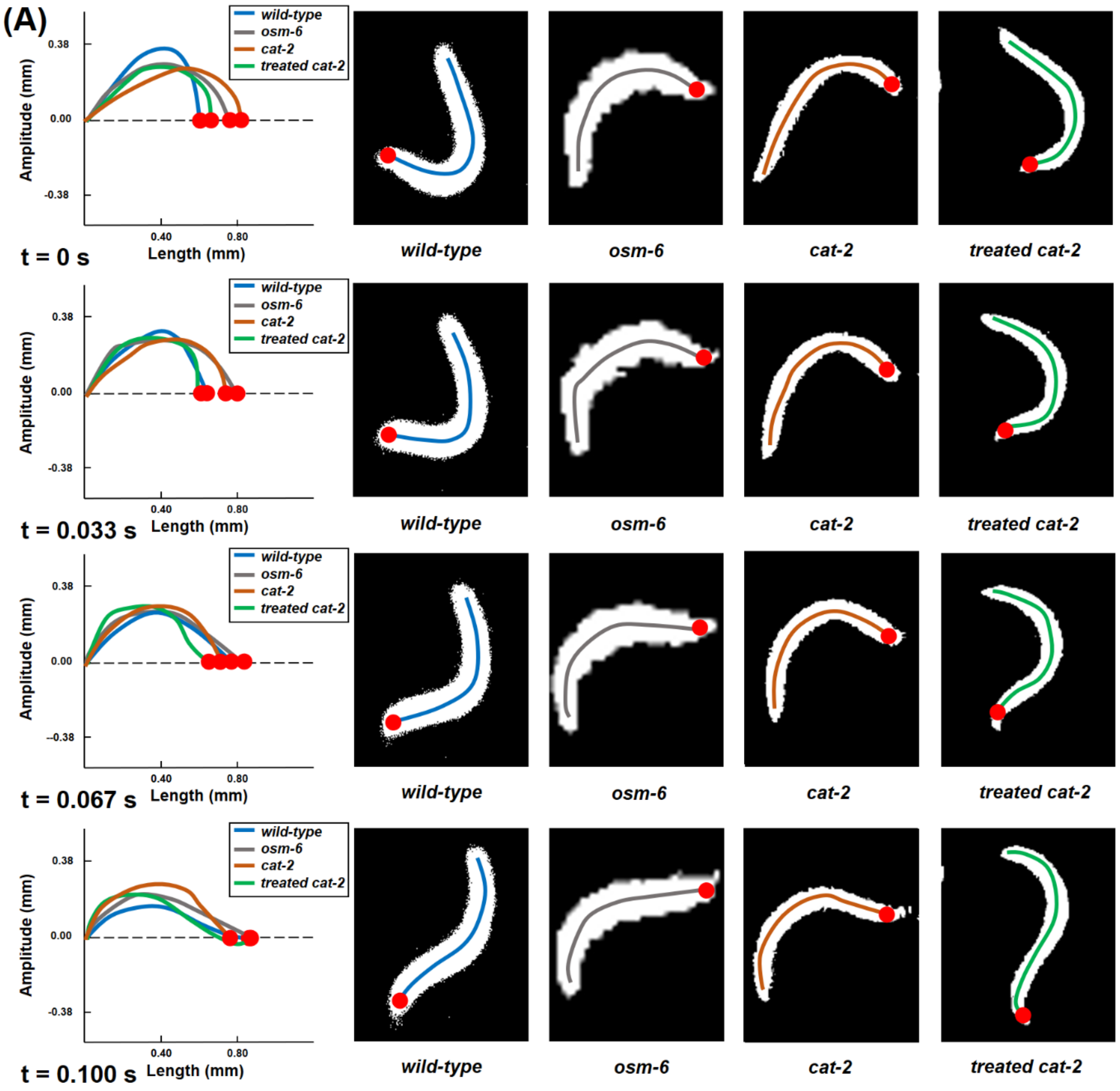

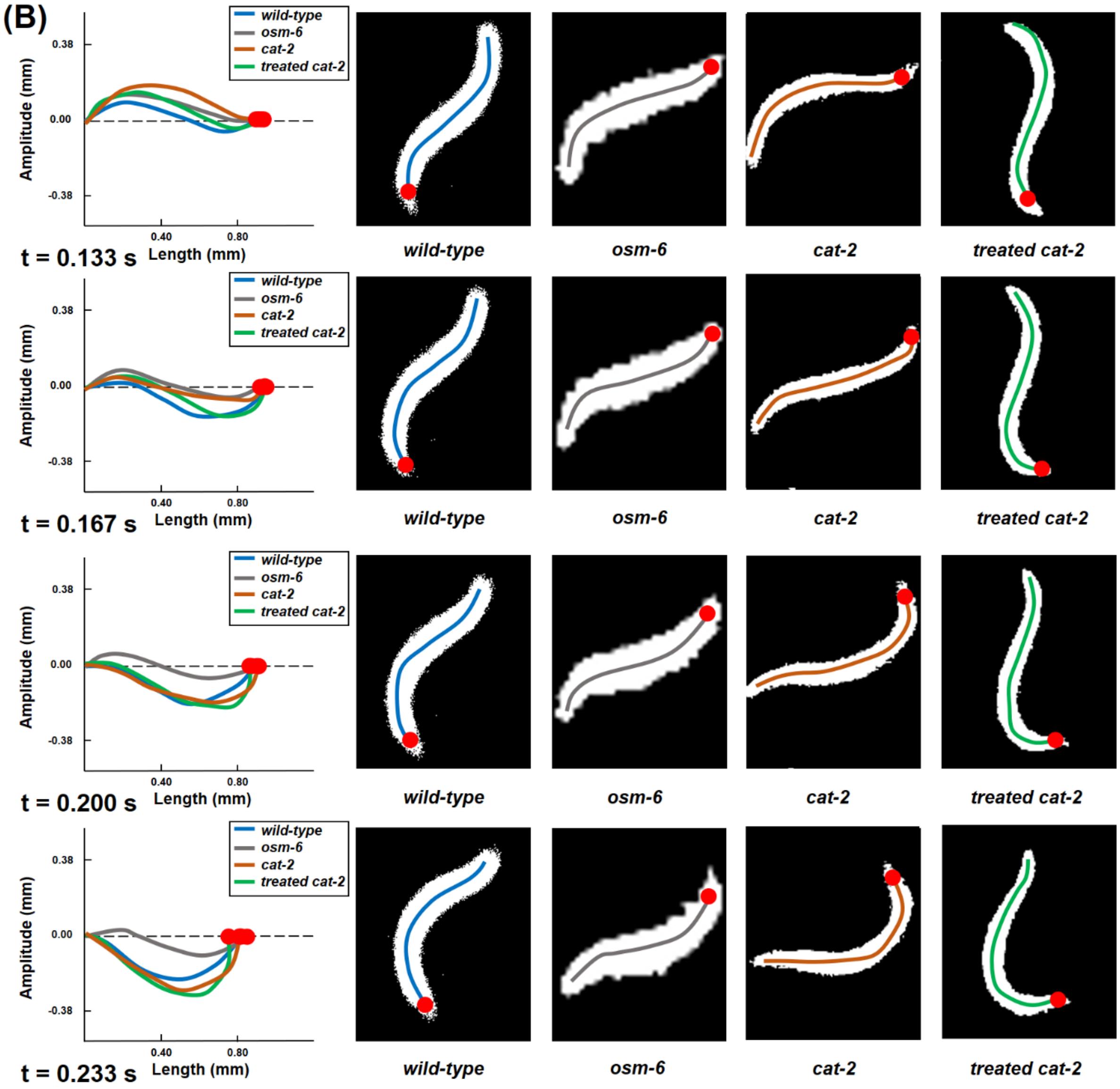

(B)
wild-type
osm-6
cat-2
treated cat-2
Amplitude (mm)
0.38
0.00
-0.38
0.40
0.80
Length (mm)
t = 0.133 s
t = 0.167 s
t = 0.200 s
t = 0.233 s
wild-type
osm-6
cat-2
treated cat-2

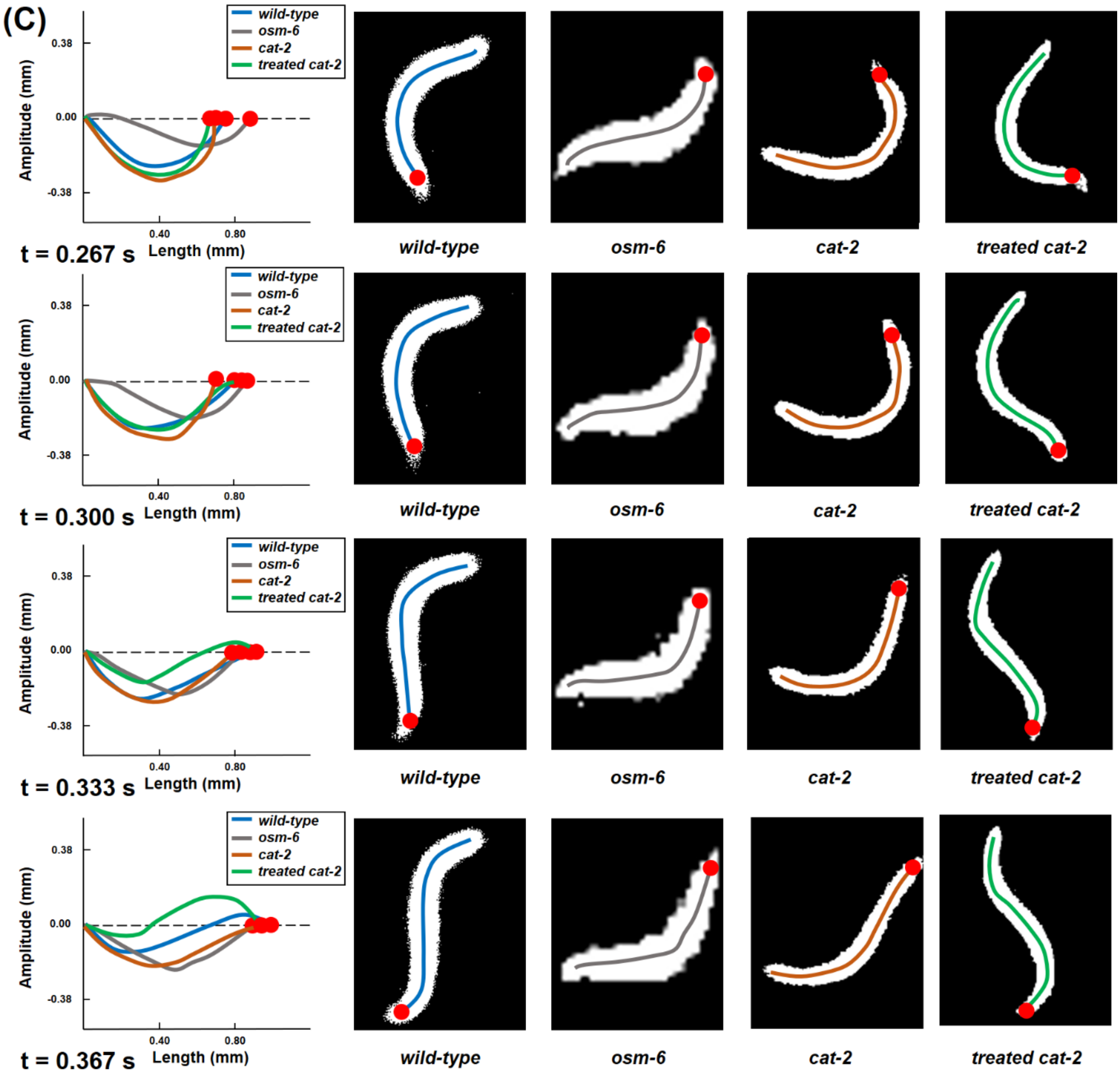
(C)
wild-type
osm-6
cat-2
treated cat-2
Amplitude (mm)
0.38
0.00
-0.38
0.40
0.80
Length (mm)
t = 0.267 s
t = 0.300 s
t = 0.333 s
t = 0.367 s

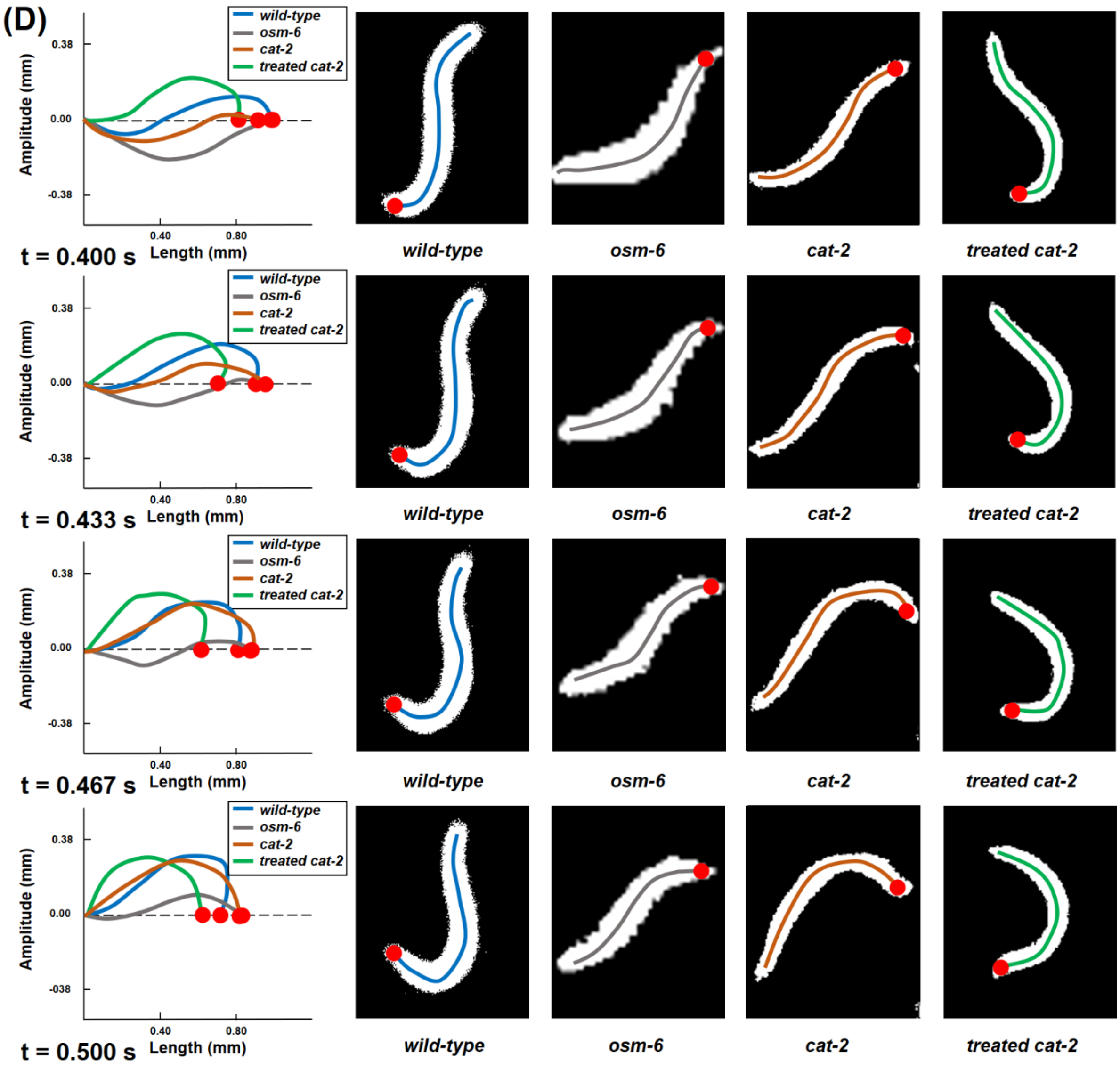
(D)
wild-type
osm-6
cat-2
treated cat-2
Amplitude (mm)
0.38
0.00
-0.38
0.40
0.80
Length (mm)
t = 0.400 s
wild-type
osm-6
cat-2
treated cat-2
t = 0.433 s
wild-type
osm-6
cat-2
treated cat-2
t = 0.467 s
wild-type
osm-6
cat-2
treated cat-2
-038
t = 0.500 s
wild-type
osm-6
cat-2
treated cat-2

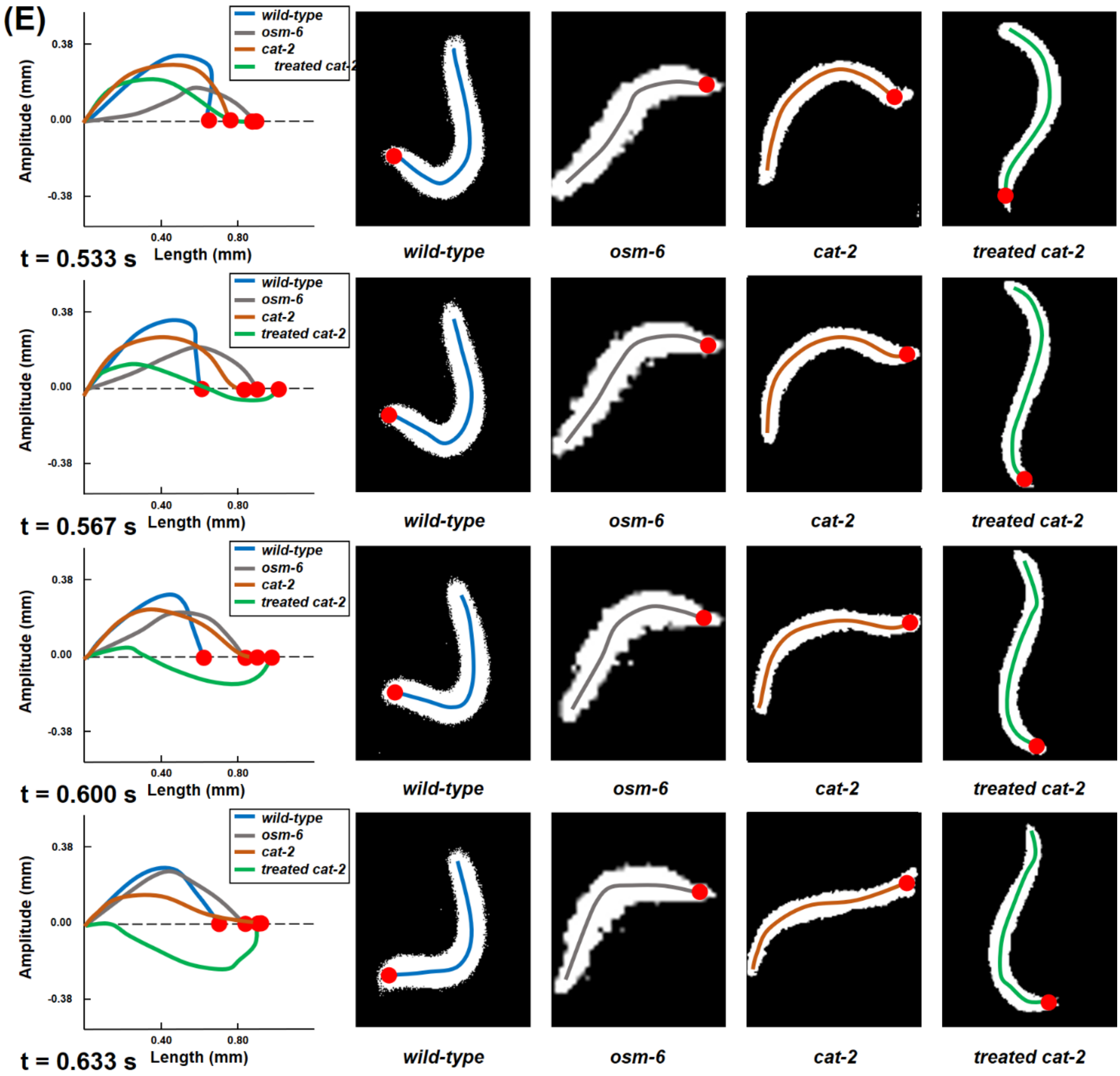

Fig. S22: The body shapes of WT, *osm-6*, *cat-2*, and pharmaceutical treated *cat-2* as functions of time t, where $0 < t < 0.6$ s. Left column: the instantaneous body centroid depicted as a function of position along the chord that connects the animal's head and tail. Right column: raw images.

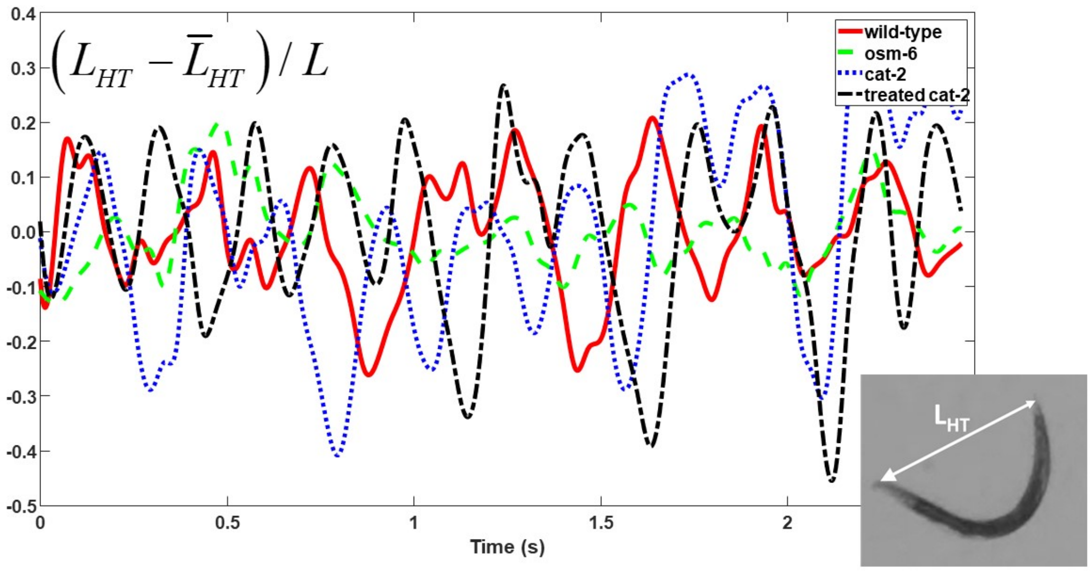

Fig. S23: $(L_{HT}-\bar{L}_{HT})/L$ of WT, *osm-6*, *cat-2*, and pharmaceutical treated *cat-2* as functions of time. L_{HT} is the distance between head and tail (defined in the inset). L is the animal's body length. $\bar{L}_{HT}$ is the average distance between head and tail.

Strain	Frequency (Hz)
WT	1.6 - 2.1
osm-6	1.6 – 2.2
cat-2	1.6 – 2.1
cat-2 (pharmaceutically treated)	1.7 – 2.1

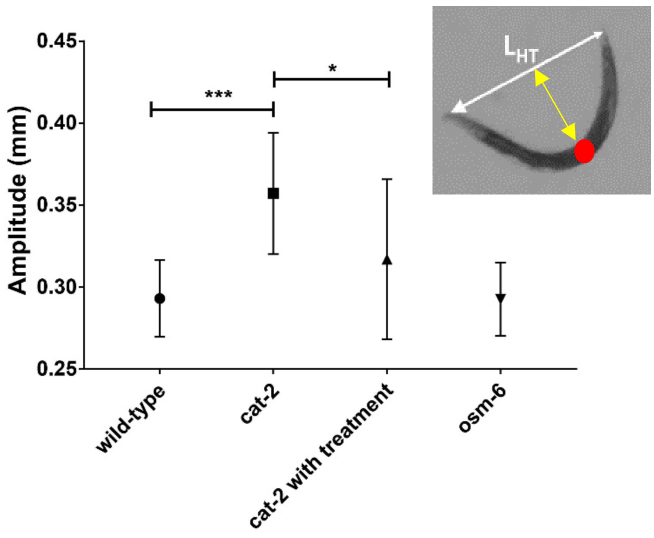

Fig. S24: Body bending frequency (left) and amplitude (right) of WT, *osm-6*, *cat-2*, and pharmaceutical treated *cat-2* (N=30). The amplitude is defined by the largest distance between the animal's body and the chord connecting head to tail (yellow arrow in the inset) (p ≤ 0.05 *, p ≤ 0.001 ***). The frequency is half the frequency determined by Fast Fourier Transform (FFT) of the time series of **Fig. S23**.

We collected images of settling WT, *osm-6*, *cat-2*, and pharmaceutical treated *cat-2* as functions of time while the animals were within the field of view of the camera (**Fig. S24**, right). These images were processed to identify the central line of each animal. The left column (**Fig. S22**) depicts the "body shape" as a function of position along the chord that connects head and tail. We inferred the animals' normalized body deformation $\left(L_{HT}-\bar{L}_{HT}\right)/L$ as functions of time (**Fig. S23**) and the animals' body bending frequency (full cycle) and amplitude (**Fig. S24**). L_{HT} is the distance between head and tail (defined in the inset in **Fig. S23**). L is the animal's body length. $\bar{L}_{HT}$ is the average distance between head and tail. The frequency of $\left(L_{HT}-\bar{L}_{HT}\right)/L$ is twice the beating frequency. Although the animals' gaits are not identical, differences in the gaits are relatively small and unlikely to explain why certain animals gravitax while others do not.

S15. Well-fed adult worms of the AB1 Adelaide strain align their direction of swimming with the direction of the gravity vector.

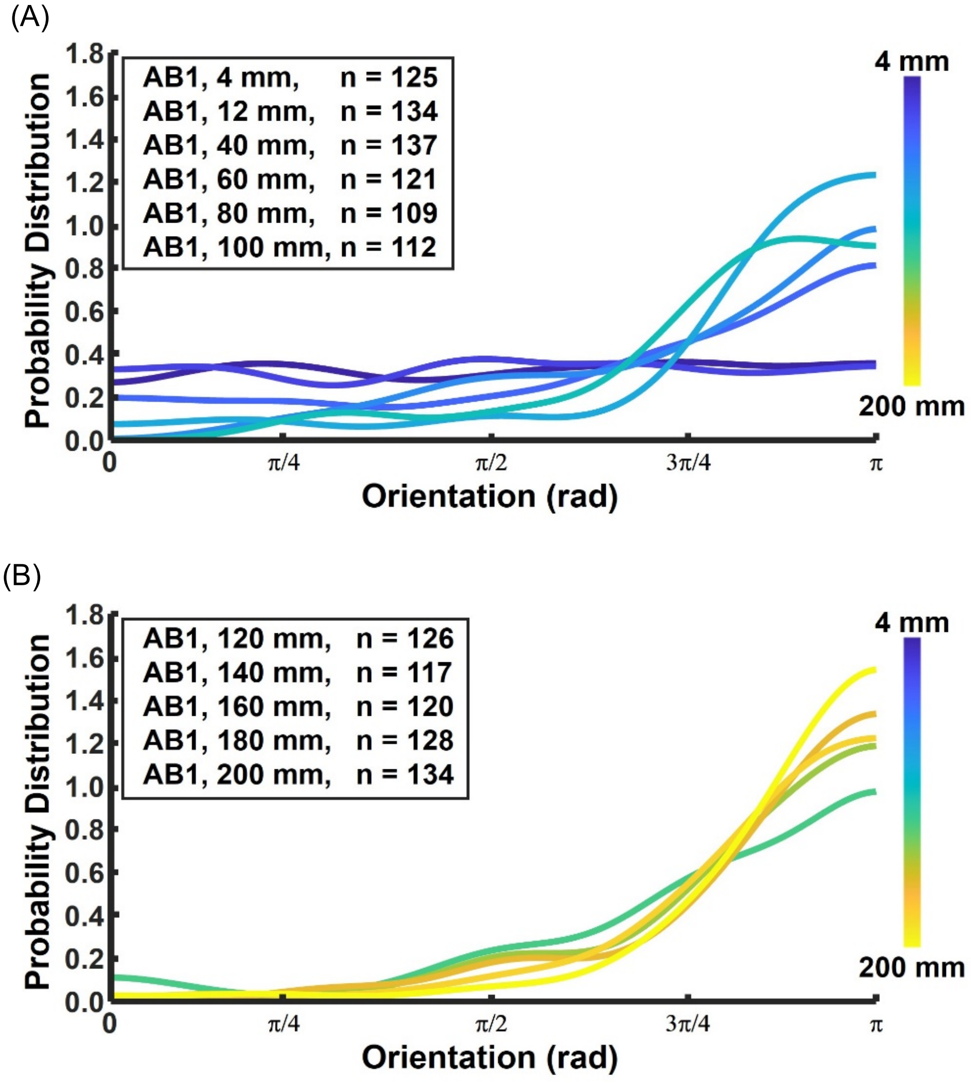

Fig. S25: **Well-fed adult worms of the AB1 Adelaide strain align their direction of swimming with the direction of the gravity vector.** (**A**) Kernel Density Estimate (KDE) AB1 mutants at depths ranging from 4 to 100 mm beneath the liquid surface. The bandwidth of the KDE smoothing window is π/12. (**B**) Kernel (probability) Density Estimate AB1 mutants at depths ranging from 120 to 200 mm beneath the liquid surface. The bandwidth of the KDE smoothing window is π/12.

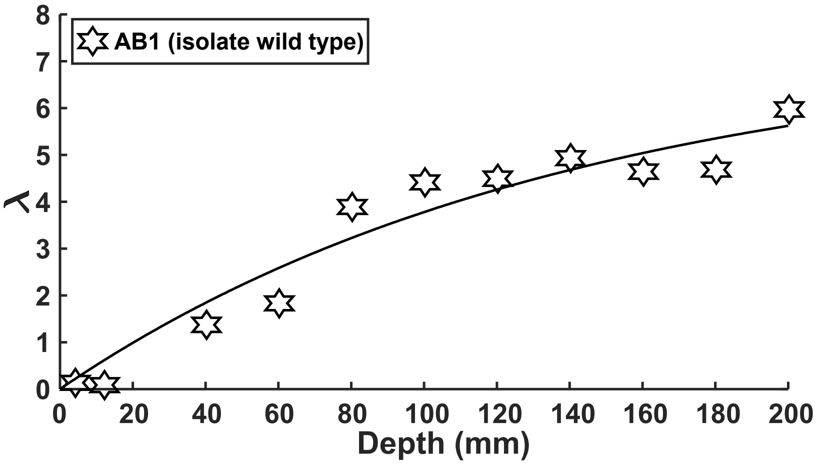

Fig. S26: The concentration factor of well-fed AB1 animals increases as their submersion depth (residence time) increases. The increase in the concentration factor indicates alignment with the direction of the gravity vector. The solid line is a best fit curve (equation 3) with λ_{∞} ~ *7.37* and β~ *0.007* mm^{-1}.

S16: *C. elegans* on agar does not gravitax

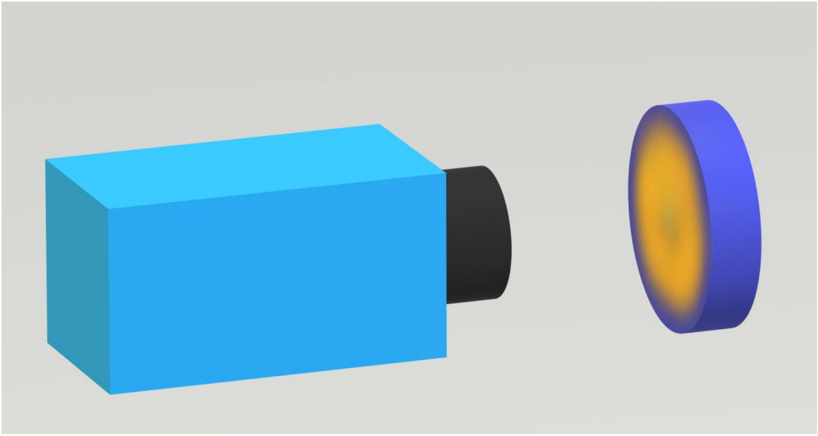

Fig. S27: The experimental set-up for monitirng the orientation of *C. elegans* on a 35 mm diameter, vertical agar plate. The camera focuses on the center of the agar plate. The width and height of the imaged area are respectively 10.4mm and 8.2mm. In the well-fed experiments, the agar was seeded with bacteria.

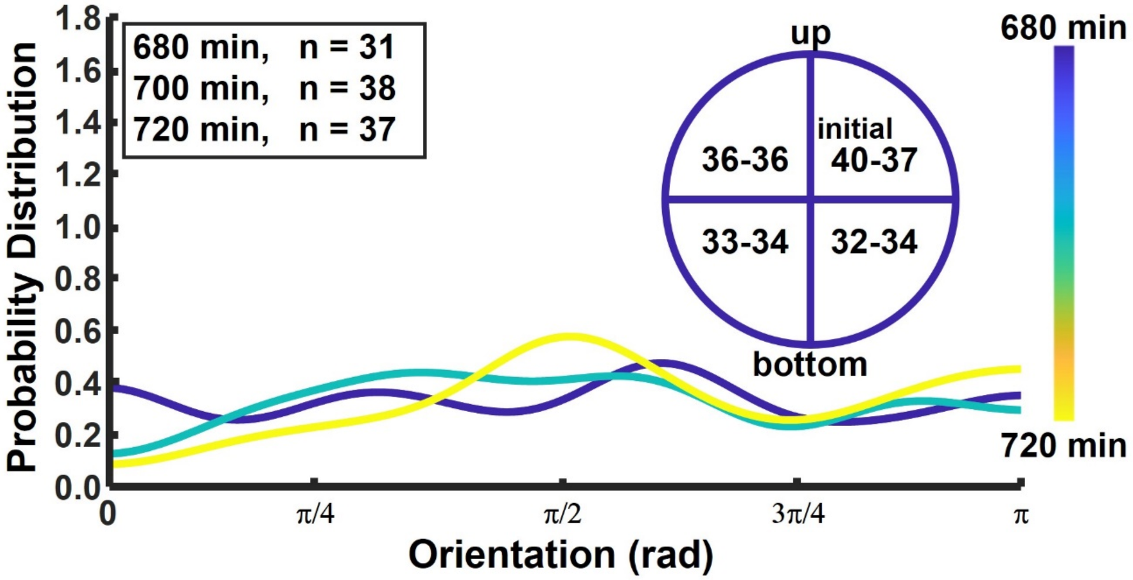

Fig. S28: Well-fed WT animals on vertical agar plate do not show gravitaxis. The Kernel-density plot (KDE) of WT orientation after 680, 700, and 720 minutes does not differ significantly from similar KDE plots at earlier times. The inset counts the number of animals in each quadrant at the beginning (time zero) and the end (after 12 hours) of the experiment.

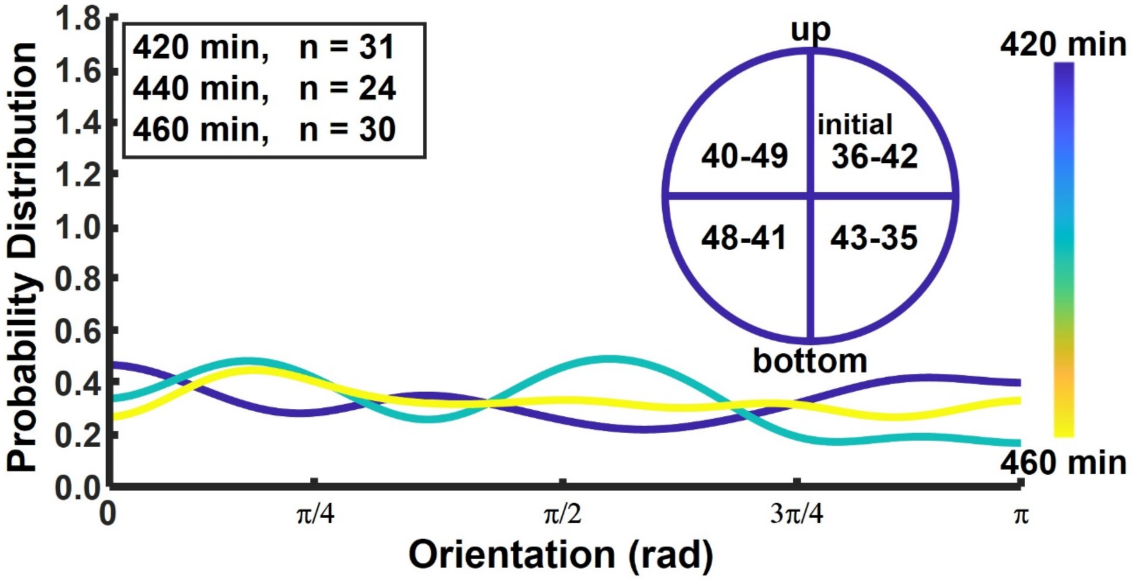

Fig. S29: Well-fed *mec-3* animals on vertical agar plate do not show gravitaxis. The Kernel-density plot (KDE) of *mec-3* orientation after 420, 440, and 480 minutes does not differ significantly from similar KDE plots at earlier times. The inset counts the number of animals in each quadrant at the beginning (time zero) and the end (after 12 hours) of the experiment.

S17. Top heavy cylinders settle broad side.

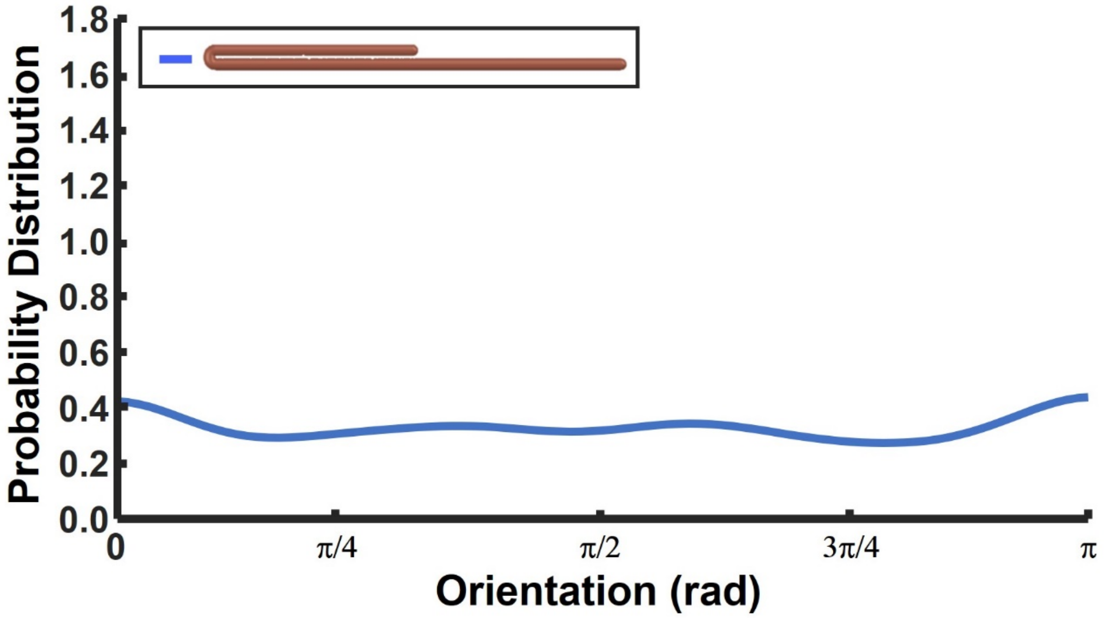

Fig. S30: Kernel (probability) Density Estimate (KDE) of the inclination angle θ of a settling top heavy metal (copper) wire of 1 mm length and 100 μm diameter folded upon itself.

To demonstrate, in principle, that non-uniform mass distribution does not necessarily cause worms to rotate and align with the direction of gravity, we carried out a simple experiment wherein we monitored the orientation of a folded copper wire settling in aqueous solution. Although the wire is heavier on one side than the other, it settled *broadside* and did not rotate to align with the direction of gravity. The metal wire is heavier and settles at higher speed than the nematode, but the mass imbalance and the torque acting to bring the wire to a vertical posture are also much greater than for the nematode.

Table S1: Summary of Our Experiments

Strain and Condition*	Characteristics	Gravitaxis?
**Worms suspended in an aqueous solution**		
N2	Wild type isolated in the Northern hemisphere	YES**
N2 without food	N2 starved	YES
N2, heat-killed	Permanent paralysis	NO
N2, sodium azide	Reversible paralysis	NO
unc-54(e190)	Severely Impaired body movement	NO
unc-29(e1072)	Mildly impaired body movement	YES
AB1	Wild type isolated in the Southern Hemisphere	YES
osm-6(p811)	Globally disrupted sensory cilia	NO
che-2(e1033)	Globally disrupted sensory cilia	NO
mec-3(e1338)	Defective response to light and harsh touch	YES
mec-4(u253)	Defective response to light touch	YES
cat-2(e1112)	Defective dopamine biosynthesis	NO
cat-2(n4547)	Defective in dopamine biosynthesis	NO
cat-2(e1112), dopamine	*cat-2* mutants pharmacologically treated with dopamine	YES
dop-1(vs101)	Defective dopamine receptor	NO
dop-2(vs105)	Defective dopamine receptor	NO
dop-3(vs106)	Defective dopamine receptor	NO
**Worms on an agar surface**		
N2	Wild type	NO
N2, without food	N2 starved	NO
mec-3(e1338)	Defective response to light and harsh touch	NO
mec-3(e1338) without food	Defective response to light and harsh touch	NO

Table S1: Gravitaxis as a function of strain and condition.

*All worms were cultivated with ample ad libitum food (i.e., well-fed) unless indicated otherwise.

** $\lambda>1$

Table S2: Percent of worms that were censored

Strain	Censored Inactive worms (Presumed dead)
**Worms suspended in an aqueous solution**	**Percentage**
N2	~ 3 %
N2 without food	~ 4 %
unc-29(e1072)	~ 17 %
AB1	~ 3 %
osm-6(p811)	~ 5 %
che-2(e1033)	~ 4 %
mec-3(e1338)	~ 7 %
mec-4(u253)	~ 5 %
cat-2(e1112)	~ 3 %
cat-2(n4547)	~ 6 %
cat-2(e1112), dopamine	~ 4 %
dop-1(vs101)	~ 3 %
dop-2(vs105)	~ 8 %
dop-3(vs106)	~ 3 %

Table S2: Fraction of censored animals in experiments with motile animals as a function of strain. Non-moving animals were censored and not included in the data analysis.

Table S3: Statistical Analysis

We tested the likelihood of the null hypothesis H_0^{WT} that any of the distributions is like that of the wild type (N2) and the null hypothesis $H_0^{Paralyzed}$ that any of the distributions is like that of the heat -paralyzed animals at approximately the same depth.

**Strain and Condition***	**Gravitaxis?**	**Probability of H_0^{WT}**	**Probability of $H_0^{Paralyzed}$**
N2	YES	1	<0.001
N2 without food	YES	0.15	<0.001
N2, heat-killed	NO	<0.001	1
N2, sodium azide*	NO	<0.001	0.85
*unc-54(e190)**	NO	<0.001	0.99
unc-29 (e1072)	YES	<0.001	<0.001
AB1	YES	0.66	<0.001
*osm-6 (p811)**	NO	<0.001	0.02
*che-2 (e1033)**	NO	<0.001	0.55
mec-3 (e1338)	YES	0.83	<0.001
mec-4 (u253)	YES	0.03	<0.001
*cat-2 (e1112)**	NO	<0.001	0.54
*cat-2 (n4547)**	NO	<0.001	0.94
cat-2 (e1112), dopamine*	YES	0.32	<0.001
*dop-1(vs101)**	NO	<0.001	0.47
*dop-2(vs105)**	NO	<0.001	0.55
*dop-3(vs106)**	NO	<0.001	0.59

* Indicates that the datasets were compared at 40 mm beneath the surface. All others were at 200 mm beneath the liquid surface.
